# Organic Ligand-Mediated Dissolution and Fractionation of Rare-Earth Elements (REEs) from Carbonate and Phosphate Minerals

**DOI:** 10.1021/acsearthspacechem.4c00009

**Published:** 2024-04-25

**Authors:** Yinghao Wen, Pan Liu, Qian Wang, Simin Zhao, Yuanzhi Tang

**Affiliations:** School of Earth and Atmospheric Sciences, Georgia Institute of Technology, 311 Ferst Drive, Atlanta, Georgia 30332, United States

**Keywords:** rare-earth elements, organic ligands, siderophore, complexation, fractionation, thermodynamic modeling

## Abstract

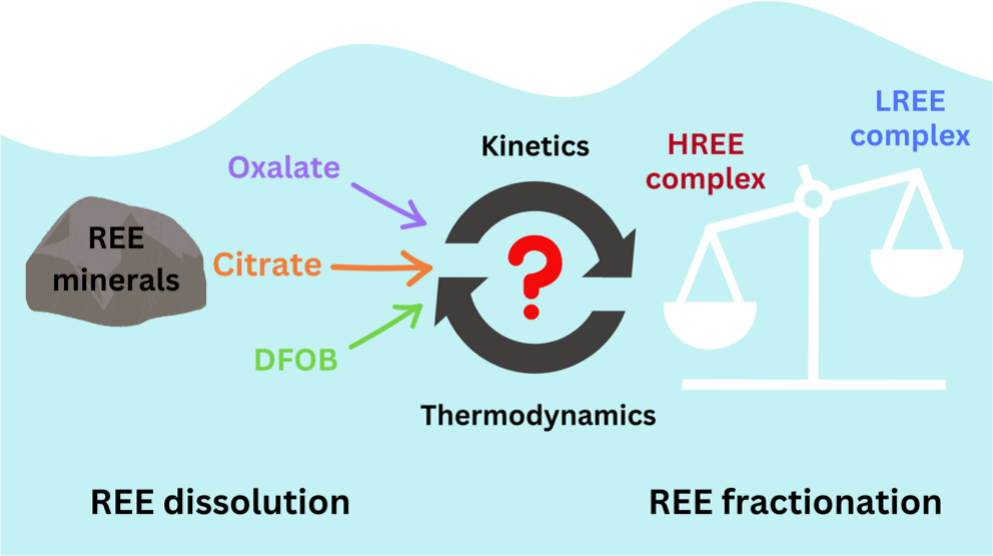

Global efforts to build a net-zero economy and the irreplaceable roles of rare-earth elements (REEs) in low-carbon technologies urge the understanding of REE occurrence in natural deposits, discovery of alternative REE resources, and development of green extraction technologies. Advancement in these directions requires comprehensive knowledge on geochemical behaviors of REEs in the presence of naturally prevalent organic ligands, yet much remains unknown about organic ligand-mediated REE mobilization/fractionation and related mechanisms. Herein, we investigated REE mobilization from representative host minerals induced by three representative organic ligands: oxalate, citrate, and the siderophore desferrioxamine B (DFOB). Reaction pH conditions were selected to isolate the ligand-complexation effect versus proton dissolution. The presence of these organic ligands displayed varied impacts, with REE dissolution remarkably enhanced by citrate, mildly promoted by DFOB, and showing divergent effects in the presence of oxalate, depending on the mineral type and reaction pH. Thermodynamic modeling indicates the dominant presence of REE–ligand complexes under studied conditions and suggests ligand-promoted REE dissolution to be the dominant mechanism, consistent with experimental data. In addition, REE dissolution mediated by these ligands exhibited a distinct fractionation toward heavy REE (HREE) enrichment in the solution phase, which can be mainly attributed to the formation of thermodynamically predicted more stable HREE–ligand complexes. The combined thermodynamic modeling and experimental approach provides a framework for the systematic investigation of REE mobilization, distribution, and fractionation in the presence of organic ligands in natural systems and for the design of green extraction technologies.

## 1 Introduction

Rare-earth elements (REEs), which include Sc, Y, and the 15 lanthanides, have been identified as “critical minerals” by the United States and the European Union due to their skyrocketing demand in clean-energy products and potential supply chain disruptions.^1,2^ In order to build a more resilient supply chain, the U.S. is making significant efforts toward establishing reliable domestic supplies.^3^ Since REEs rarely form concentrated ore deposits, geological constraints of REE mining and the need to diversify REE resources have led to surging interests in the exploration of geological deposits, recovery of REEs from waste resources, and development of green extraction technologies.^4−7^ In addition to the heavy REE (HREE) reserves in southeast China (currently accounting for ∼80% of global HREE supply), explored natural deposits include highly weathered environments in Madagascar, Brazil, southeastern U.S., and southeast Asia.^4^ Studies have also investigated the recovery of REEs from solid wastes such as coal fly ash,^8^ municipal solid waste incineration ash,^9^ spent magnets,^10^ and phosphogypsum.^11^

Exploration in these directions requires a systematic understanding of the mobilization and fractionation of REEs from host solids under natural or engineered settings that involve common inorganic/organic complexing ligands.^12,13^ In natural waters, dissolved REEs primarily exist as free cations or chloride/sulfate complexes under acidic conditions, whereas carbonate complexes are dominant in the neutral-to-alkaline pH range.^14−16^ In addition to inorganic ligands, REE mobilization from minerals are also strongly influenced by organic ligands in natural environments via ligand-promoted dissolution by forming soluble REE–ligand complexes.^12,17−20^ Elevated REE concentrations in natural waters and sediments are often observed to be associated with organics. A field study showed a positive linear relationship between the total REE concentration in stream waters and the dissolved organic carbon (DOC) level, suggesting the formation of organic–REE complexes.^24^ Moreover, dissolved REE patterns in natural waters generally display a noticeable fractionation toward HREE enrichment.^21−24^ Yet, a mechanistic understanding of REE interaction with different organic ligands remains lacking.^12,21,25^ To better understand the impacts of organic ligands on REE mobilization and fractionation, several controlled laboratory studies were conducted using representative natural organic ligands. Enhanced REE dissolution from apatite and monazite has been reported in the presence of low-molecular-weight organic acids (e.g., citrate and phthalate) at low concentrations.^26^ Similar results were observed on granite at near-neutral pH.^27^ Siderophores, a group of Fe(III)-chelating organic ligands, were shown to promote REE dissolution from volcanic ash and lead to REE fractionation with LREE depletion, middle REE enrichment, and a positive Ce anomaly.^28^ Similar phenomena have been observed in the Amazon River and western Pacific Ocean, suggesting REE complexation with organic ligands to be one of the key processes governing REE mobilization and transport in natural aquatic systems.^29,30^ On the other hand and from the engineering perspective, conventional hydrometallurgy extraction methods typically use strong mineral acids (e.g., hydrochloric acid) for REE extraction from solid phases.^31,32^ Recent studies have explored the design of green technologies using organic ligands for REE extraction from solid wastes such as spent magnet and municipal solid waste incineration ash.^9,10^

Despite the aforementioned studies on organic ligand-mediated REE dissolution and fractionation, much critical information remains largely missing, including (1) the kinetics of REE dissolution in the presence of organic ligands; (2) whether current thermodynamic data (e.g., solubility of REE minerals and stability constants of REE–organic complexes) can predict the impact of organic ligands on REE fate and transport; and (3) major driving forces of REE fractionation patterns and dominant species of REE–ligand complexes. Answers to these questions carry profound significance from both geological and environmental perspectives. Such knowledge can enable further investigation on many geological/geochemical processes relying on the REE system as tracers and analogues.^12,33^ In addition, an in-depth understanding of organic ligand-mediated REE behaviors can contribute to the exploration of potential REE deposits and the design of efficient ligand-based technologies for REE separation and purification.

This study aims to address the aforementioned knowledge gaps by investigating REE dissolution and fractionation from common host minerals in the presence of naturally occurring organic ligands: citrate, oxalate, and the siderophore desferrioxamine B (DFOB). Since REE carbonate and phosphate minerals and their altered/weathered derivatives account for the majority of global REE reserve, they were selected to represent REE host minerals.^17,34−36^ We note that rare-earth elements typically co-occur with each other due to their similar chemical properties. In this study, we use minerals containing a single rare-earth element in order to facilitate the constraint of reaction mechanisms within experimental groups and cross-comparison with thermodynamic modeling. Such an experimental design also allows the interpretation of fractionation patterns without interference from kinetic constraints in experiments involving multiple rare-earth elements (e.g., preferential leaching, diffusion limitation, and particle size effects). La and Nd were selected as representative light REEs (LREEs), while Y was chosen as a probe for HREEs due to similar chemical properties (e.g., complexation, local coordination) and relatively high natural abundance.^37^ Oxalate and citrate are ubiquitous organic ligands in soils and water bodies and have high affinities toward REE complexation (log β of ∼6.0–7.4 and ∼9.1–11.2, respectively).^24,38^ Citrate can be produced by various microorganisms and is commonly present in rhizosphere solutions (∼0.6–3.1 μM), whereas oxalate is one of the most abundant dicarboxylic ligands in aquatic sediments and aerosols (0.1–0.7 mM).^39−41^ Siderophores are one important group of biogenic organic chelating ligands that are released by many microbes (both bacteria and fungi) to scavenge Fe(III) from low-solubility Fe(III)-minerals.^42^ Siderophores were also previously shown to have strong affinity toward other trivalent metal cations such as Cr(III), Mn(III), and Co(III) due to their structural similarity to Fe(III).^43−45^ DFOB is the most studied model siderophore to date and was reported to outcompete carbonate (the dominant inorganic REE-complexing ligand in seawater) for REE complexation due to the high stability constants of REE–DFOB complexes (log β ≈ 10.1–15.2).^46^ Note that organic ligands have been known to mediate metal dissolution from solid phases via both ligand-promoted and proton-promoted mechanisms depending on the solution pH. Our previous work reported that proton-induced dissolution of REE carbonates (e.g., tengerite and bastnäsite) occur at ∼pH 6 and lower, whereas REE phosphates (e.g., rhabdophane and monazite) barely dissolve at pH above 2 (Figure S1).^34^ In order to isolate the effect of ligand-promoted dissolution and eliminate the contribution from proton-promoted dissolution,^47,48^ we chose pH 8 and pH 4 for carbonate and phosphate minerals, respectively. In addition, a pH of 7 was selected for both systems to represent natural neutral conditions.

In this study, the patterns of REE dissolution and fractionation in the presence of these organic ligands at different pH values were experimentally obtained, and the corresponding REE dissolution rates were calculated. Thermodynamic calculations were conducted using published REE mineral solubility and REE–ligand complex stability constants and were combined with experimental data to elucidate the underlying reaction mechanisms and dominant speciation of REE–ligand complexes.^49^ Finally, local complexation environments of REE–ligand complexes were discussed to explain the observed fractionation pattern.

## 2 Materials and Methods

### 2.1 REE Minerals and Reagents

Unless specified otherwise, all chemicals used were ACS grade or higher (Text S1). La(III), Nd(III), and Y(III) carbonate minerals, namely, lanthanite-(La), tengerite-(Nd), and tengerite-(Y), were purchased and used as received. La(III), Nd(III), and Y(III) phosphate minerals, namely, rhabdophane-(La), rhabdophane-(Nd), and churchite-(Y), were synthesized following the literature.^50^ All REE minerals were characterized using X-ray diffraction (XRD), scanning electron microscopy (SEM), and Brunauer–Emmett–Teller (BET) surface area analysis (Text S2). Some of the minerals have been characterized in our previous studies.^34,51^ See Table S1 for details of REE minerals, XRD mineralogy, and the BET surface area. The speciation of oxalic acid (H_2_C_2_O_4_), citric acid (C_6_H_8_O_7_), and DFOB (H_4_DFOB^+^) as a function of pH is shown in Figure S2.

### 2.2 Batch Experiments on Ligand-Induced Dissolution of REE Minerals

Considering that oxalate/citrate (∼10^–5^–10^–3^ M) and DFOB (∼10^–8^–10^–4^ M) are typically present at vastly different concentrations in natural environments, their upper limits were used in this study (1 mM oxalate/citrate; 0.1 mM DFOB).^42^ REE minerals (0.5 g/L) were mixed with a 0.1 M NaCl solution containing organic ligands with constant stirring (200 rpm) at room temperature (∼23 °C). Blank control was conducted in a 0.1 M NaCl solution. The solution pH was maintained at 7.0 or 8.0 (±0.1) for the REE carbonate system and at 4.0 or 7.0 (±0.1) for the REE phosphate system by titrating using dilute HCl or NaOH solutions (Table S1). Syringe-filtered (0.2 μm, PTFE) aliquots were collected at predetermined time points and analyzed for dissolved REE concentrations using inductively coupled plasma mass spectrometry (ICP-MS; details in Text S2). All experiments were conducted in duplicate. Errors were calculated by dividing the standard deviation by the square root of number of replicates. No microbial growth was observed.

To obtain the initial REE dissolution rates, a regression line was fitted through the linear range (the first 2 h for REE carbonate minerals and 6 h for REE phosphate minerals) of the time profiles of dissolved REE concentrations, and the slope was normalized by the BET surface area of each mineral. Dissolved REE concentrations at the steady state were measured at 24 h for REE carbonates and 48 h for REE phosphates. A longer reaction time was used to achieve equilibrium for the dissolution of REE phosphates due to slower kinetics.

### 2.3 Thermodynamic Modeling: General Approach

To better understand REE speciation and to predict the extent of REE dissolution in the presence of organic ligands, thermodynamic calculations were conducted using the software PHREEQC version 3.^49^ Stability constants of REE–ligand complexes (β^0^) and solubility products of REE minerals (*K*_sp_^0^) at zero ionic strength (infinite dilution, *I* = 0), 25 °C, and 1 bar were compiled from the literature.^24,46,52−61^ Complexes of REE and common inorganic ligands that were considered in PHREEQC modeling include REE(Cl)^2+^, REE(OH)^2+^, REE(HCO_3_)^2+^, REE(CO_3_)^+^, REE(CO_3_)_2_^–^, REE(H_2_PO_4_)^2+^, REE(HPO_4_)^+^, REE(HPO_4_)_2_^–^, REE(PO_4_)^0^, and REE(PO_4_)_2_^3–^, where REE^3+^ represents an individual trivalent REE cation. The considered REE minerals include REE hydroxides [REE(OH)_3_],^60^ REE carbonates [REE_2_(CO_3_)_3_],^55^ REE phosphates [REE(PO_4_)],^59^ and REE oxalates [REE_2_(C_2_O_4_)_3_].^61^

Important REE–oxalate and REE–DFOB complexes include REE(C_2_O_4_)^+^, REE(C_2_O_4_)_2_^–^,^26^ REE(HDFOB)^+^, REE(H_2_DFOB)^2+^, and REE(H_3_DFOB)^3+^.^46^ Previous studies have proposed various forms of REE–citrate complexes with different stability constants,^24^ as summarized in Table S2. Considering the complexity and lack of consensus in the species of REE–citrate complexes in the literature, we carefully reviewed previous studies and selected four REE–citrate complexes that were most commonly proposed, namely, REE(C_6_H_5_O_7_)^0^, REE(C_6_H_5_O_7_)_2_^3–^, REE(C_6_H_6_O_7_)^+^, and REE(C_6_H_5_O_7_)(C_6_H_6_O_7_)^2–^ as the major REE-citrate species considered in PHREEQC modeling. Literature β^0^ values were compiled and used in this study.^56−58^ More details are discussed in the results section of citrate.

### 2.4 Thermodynamic Modeling: Ionic Strength Correction for the Stability Constants of REE–Ligand Complexes

PHREEQC thermodynamic calculations were conducted based on the logarithm of stability constants, log(β^0^), at zero ionic strength (*I* = 0) and 25 °C. However, the log(β) of REE–citrate and REE–DFOB complexes in literature were reported at ionic strengths of 0.1 ≤ *I* ≤ 1 M.^46,56−58^ Hence, we converted these log(β) to log(β^0^) using methods described as follows. Considering a general reaction for REE–ligand formation
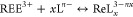
1where REE^3+^, L^*n*–^, and REEL_*x*_^3–*nx*^ represent the trivalent REE cation, negatively charged ligand, and REE–ligand complex, respectively. The stability constants β^0^ can be written as
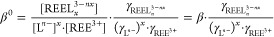
2where the values in the brackets are concentrations of each species, γ is the activity coefficient, and β is the measured stability constant at *I*.^24,52,53,62,63^ The logarithm of eq 2 is shown in eq 3
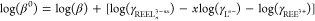
3The Davies equation was then used to obtain γ eq 4, where *z_i_* is the charge of the species. The Davies equation has been widely used for ionic strength correction with reasonable estimation.^24,52,62,64^
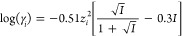
4Based on the Davies equation, eq 3 can be rewritten as eq 5
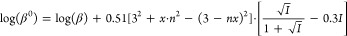
5Finally, log(β^0^) could be calculated based on log(β) and *I*. The reported log(β) and calculated log(β^0^) values for REE–citrate and REE–DFOB complexes are summarized in Table 1. Note that the approximation of log(β^0^) values using the Davies equation may add to uncertainty in thermodynamic modeling results.

**Table 1:** Logarithm of the Stability Constants of REE–Ligand Complexes (log β^0^) and the Solubility Products of REE Minerals (log *K*_sp_^0^) at Zero Ionic Strength (*I* = 0) and 25 °C Obtained from the Literature^24,46,52−61^^a^

reaction equations	note	La	Ce	Pr	Nd	Sm	Eu	Gd	Tb	Dy	Ho	Y	Er	Tm	Yb	Lu
REE^3+^ + Cl^–^ ⇌ REE(Cl)^2+^	log(β^0^)	0.65	0.65	0.65	0.65	0.65	0.65	0.65	0.65	0.65	0.65	0.65	0.65	0.65	0.65	0.65
REE^3+^ + OH^–^ ⇌ REE(OH)^2+^	log(β^0^)	5.19	5.66	5.68	5.82	6.16	6.24	6.17	6.36	6.41	6.44	6.20	6.48	6.61	6.76	6.73
REE^3+^ + HCO_3_^–^ ⇌ REE(HCO_3_)^2+^	log(β^0^)	2.34	2.31	2.25	2.28	2.34	2.47	2.36	2.46	2.50	2.46	2.32	2.49	2.52	2.53	2.49
REE^3+^ + CO_3_^2–^ ⇌ REE(CO_3_)^+^	log(β^0^)	6.73	7.06	7.23	7.28	7.46	7.48	7.39	7.46	7.56	7.55	7.48	7.61	7.68	7.81	7.75
REE^3+^ + 2CO_3_^2–^ ⇌ REE(CO_3_)_2_^–^	log(β^0^)	11.30	11.76	12.08	12.17	12.53	12.63	12.48	12.78	12.91	13.00	12.63	13.12	13.27	13.30	13.37
REE^3+^ + H_2_PO_4_^–^ ⇌ REE(H_2_PO_4_)^2+^	log(β^0^)	2.50	2.50	2.45	2.40	2.35	2.40	2.40	2.40	2.40	2.30	2.40	2.40	2.50	2.40	2.50
REE^3+^ + HPO_4_^2–^ ⇌ REE(HPO_4_)^+^	log(β^0^)	5.10	5.20	5.40	5.40	5.60	5.70	5.70	5.80	5.80	5.80	5.90	5.90	5.90	6.00	6.00
REE^3+^ + 2HPO_4_^2–^ ⇌ REE(HPO_4_)_2_^–^	log(β^0^)	8.40	8.70	8.90	9.10	9.40	9.60	9.60	9.70	9.80	9.90	9.90	10.00	10.10	10.20	10.30
REE^3+^ + PO_4_^3–^ ⇌ REE(PO_4_)^0^	log(β^0^)	10.96	11.35	11.60	11.80	12.10	12.20	12.20	12.40	12.50	12.60	12.60	12.70	12.80	12.90	13.00
REE^3+^ + 2PO_4_^3–^ ⇌ REE(PO_4_)_2_^3–^	log(β^0^)	17.60	18.50	19.08	19.50	20.40	20.66	20.70	21.00	21.20	21.30	21.40	21.40	21.60	21.90	21.90
REE^3+^ + C_2_O_4_^2–^ ⇌ REE(C_2_O_4_)^+^	log(β^0^)	5.83	6.33	6.28	6.41	6.64	6.72	6.68	6.79	6.99	6.90		6.96	7.03	7.02	7.14
REE^3+^ + 2C_2_O_4_^2–^ ⇌ REE(C_2_O_4_)_2_^–^	log(β^0^)	9.35	10.48	10.27	10.51	11.07	11.22	11.21	11.42	11.51	11.54		11.66	11.74	11.95	11.96
REE^3+^ + C_6_H_5_O_7_^3–^ ⇌ REE(C_6_H_5_O_7_)^0^	log(β)	6.65	6.70						7.15					7.51		7.62
	log(β^0^)	8.49	8.63						9.08					9.44		9.55
REE^3+^ + 2C_6_H_5_O_7_^3–^ ⇌ REE(C_6_H_5_O_7_)_2_^3–^	log(β)		11.21						12.18					12.74		13.00
	log(β^0^)	12.90	13.14						14.11					14.67		14.93
REE^3+^ + C_6_H_6_O_7_^2–^ ⇌ REE(C_6_H_6_O_7_)^+^	log(β)	3.80	5.10						5.54					5.60		5.66
	log(β^0^)	5.02	6.39						6.83					6.89		6.95
REE^3+^ + C_6_H_5_O_7_^3–^ + C_6_H_6_O_7_^2–^ ⇌ REE(C_6_H_5_O_7_)(C_6_H_6_O_7_)^2–^	log(β)	10.03		10.74	10.90	11.14	11.11	11.05	11.19	11.13	11.21		11.32	11.43	11.77	11.82
	log(β^0^)	12.18	12.53^b^	12.89	13.05	13.29	13.26	13.20	13.34	13.28	13.36		13.47	13.58	13.92	13.97
REE^3+^ + H_3_DFOB^0^ ⇌ REE(H_3_DFOB)^3+^	log(β)	4.88		5.45	5.54	5.93	6.07	6.04	6.24	6.35	6.32	6.16	6.38	6.44	6.53	6.48
	log(β^0^)	4.88		5.45	5.54	5.93	6.07	6.04	6.24	6.35	6.32	6.16	6.38	6.44	6.53	6.48
REE^3+^ + H_2_DFOB^–^ ⇌ REE(H_2_DFOB)^2+^	log(β)	7.70		8.96	9.24	10.09	10.32	10.31	10.67	10.83	10.89	10.51	10.99	11.13	11.27	11.25
	log(β^0^)	8.45		9.71	9.99	10.84	11.07	11.06	11.42	11.58	11.64	11.26	11.74	11.88	12.02	12.00
REE^3+^ + HDFOB^2–^ ⇌ REE(HDFOB)^+^	log(β)	10.09		11.96	12.33	13.38	13.67	13.67	14.15	14.40	14.53	13.98	14.66	14.90	15.17	15.19
	log(β^0^)	11.59		13.46	13.83	14.88	15.17	15.17	15.65	15.90	16.03	15.48	16.16	16.40	16.67	16.69
REE(PO_4_)(s) ⇌ REE^3+^ + PO_4_^3–^	log(*K*_sp_^0^)	–25.75	–26.27	–26.43	–26.20	–26.19	–25.96	–25.62	–25.39	–25.18	–25.07	–25.02	–25.13	–25.03	–24.89	–24.75
REE_2_(CO_3_)_3_(s) ⇌ 2REE^3+^ + 3CO_3_^2–^	log(*K*_sp_^0^)	–35.30	–35.10	–34.80	–34.65	–34.50	–35.00	–34.70	–34.20	–34.00	–33.80	–32.80	–33.60	–33.40	–33.30	–33.00
REE(OH)_3_(s) ⇌ REE^3+^ + 3OH^–^	log(*K*_sp_^0^)	–22.29	–23.88	–24.38	–25.98	–25.87	–26.54	–26.89	–26.31	–25.90	–26.57	–25.93	–26.57	–26.75	–26.64	–26.99
REE_2_(C_2_O_4_)_3_(s) ⇌ 2REE^3+^ + 3C_2_O_4_^2–^	log(*K*_sp_^0^)	–29.22	–30.4		–30.89	–31.35	–31.38	–31.37		–30.70		–29.29	–30.04		–30.02	

a The stability constants of REE–citrate and REE–DFOB complexes reported at ionic strength *I* > 0 (noted as log(β)) were corrected to log(β^0^) using the Davies equation eq 4. REE^3+^ represents the individual trivalent REE cation.

b Estimated value based on log(β^0^) of La and Pr: log(β_Ce_^0^) = log(β_La_^0^) + log(β_Pr_^0^).

## 3 Results and Discussion

### 3.1 Characterization of REE Minerals

**Figure 1:**
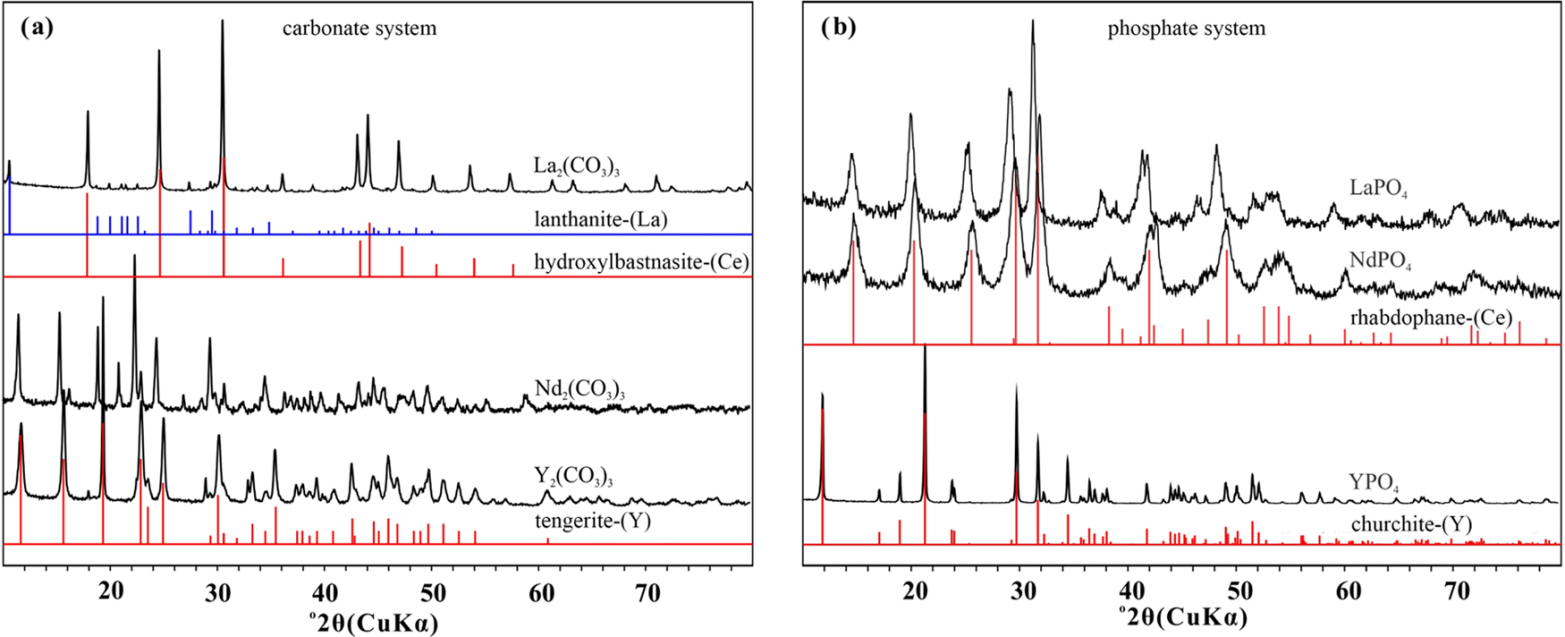
XRD patterns of REE minerals in this study. (a) REE-carbonates: La_2_(CO_3_)_3_, Nd_2_(CO_3_)_3_, and Y_2_(CO_3_)_3_; (b) REE-phosphates: LaPO_4_, NdPO_4_, and YPO_4_. Vertical lines indicate reference compounds lanthanite-(La) [(La, Nd)_2_(CO_3_)_3_·8H_2_O] (PDF #30–0678), hydroxylbastnäsite-(Ce) [CeCO_3_OH] (PDF #32–0189), tengerite-(Y) [Y_2_(CO_3_)_3_·2–3H_2_O] (PDF #24–1419), rhabdophane-(Ce) [CePO_4_·H_2_O] (PDF #35–0614), and churchite-(Y) [YPO_4_·2H_2_O] (PDF #85–1842). The reference pattern of hydroxylbastnäsite-(Ce) is shown here because it is almost identical to that of hydroxylbastnäsite-(La) except for slight shifts, and the pattern of hydroxylbastnäsite-(La) is not available.^78^ Similarly, the reference patterns of rhabdophane-(La) and rhabdophane-(Nd) are almost identical to that of rhabdophane-(Ce) and are thus not shown here.^79^

XRD analyses (Figure 1a) showed that La(III) carbonate (hereafter as La_2_(CO_3_)_3_) is mainly composed of hydroxylbastnäsite-(La) [LaCO_3_OH] with a trace amount of lanthanite-(La) [LaCO_3_OH], while Nd(III) and Y(III) carbonate minerals (hereafter as Nd_2_(CO_3_)_3_ and Y_2_(CO_3_)_3_, respectively) are identified as tengerite-(Nd) [Nd_2_(CO_3_)_3_·2–3H_2_O] and tengerite-(Y) [Y_2_(CO_3_)_3_·2–3H_2_O], respectively. For the phosphate system, the synthesized La(III), Nd(III), and Y(III) phosphates (hereafter as LaPO_4_, NdPO_4_, and YPO_4_, respectively) are identified as rhabdophane-(La) [LaPO_4_·H_2_O], rhabdophane-(Nd) [NdPO_4_·H_2_O], and churchite-(Y) [YPO_4_·2H_2_O], respectively (Figure 1b).

SEM showed that the REE minerals have a small particle size of <5 μm. La_2_(CO_3_)_3_ displays a near-spherical morphology; Nd_2_(CO_3_)_3_ particles show a rod-like shape; and Y_2_(CO_3_)_3_ is composed of flake particles (Figure S3a–c). NdPO_4_ and YPO_4_ particles have needle- and rod-like shapes, respectively, whereas LaPO_4_ occurs as irregularly shaped aggregates (Figure S3d–f). The BET specific surface areas of these minerals are around 2–7 m^2^/g, except for LaPO_4_ and NdPO_4_ that have much larger surface areas of 77 and 115 m^2^/g, respectively (Table S1).

### 3.2 REE Dissolution without Organic Ligands

**Figure 2:**
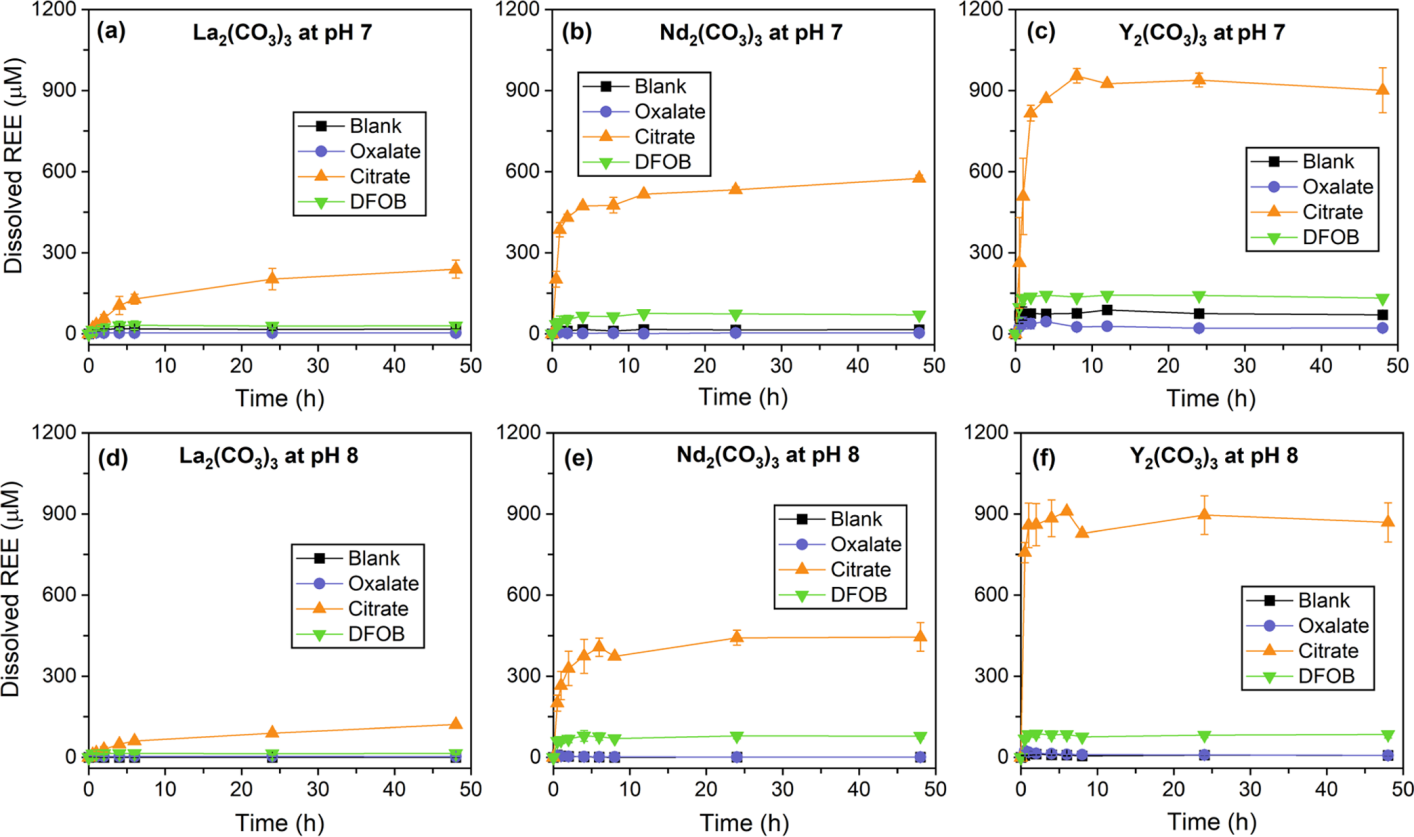
Time profiles of dissolved REEs from La_2_(CO_3_)_3_, Nd_2_(CO_3_)_3_, and Y_2_(CO_3_)_3_ at (a–c) pH 7 and (d–f) pH 8 in blank control, with 1 mM oxalate, 1 mM citrate, and 0.1 mM DFOB. Initial dissolution rates were obtained by fitting the data points during the beginning of reactions (see Figure 4 and Table S3). To compare REE behavior difference in the presence of the same ligand, see Figure S5.

**Figure 3:**
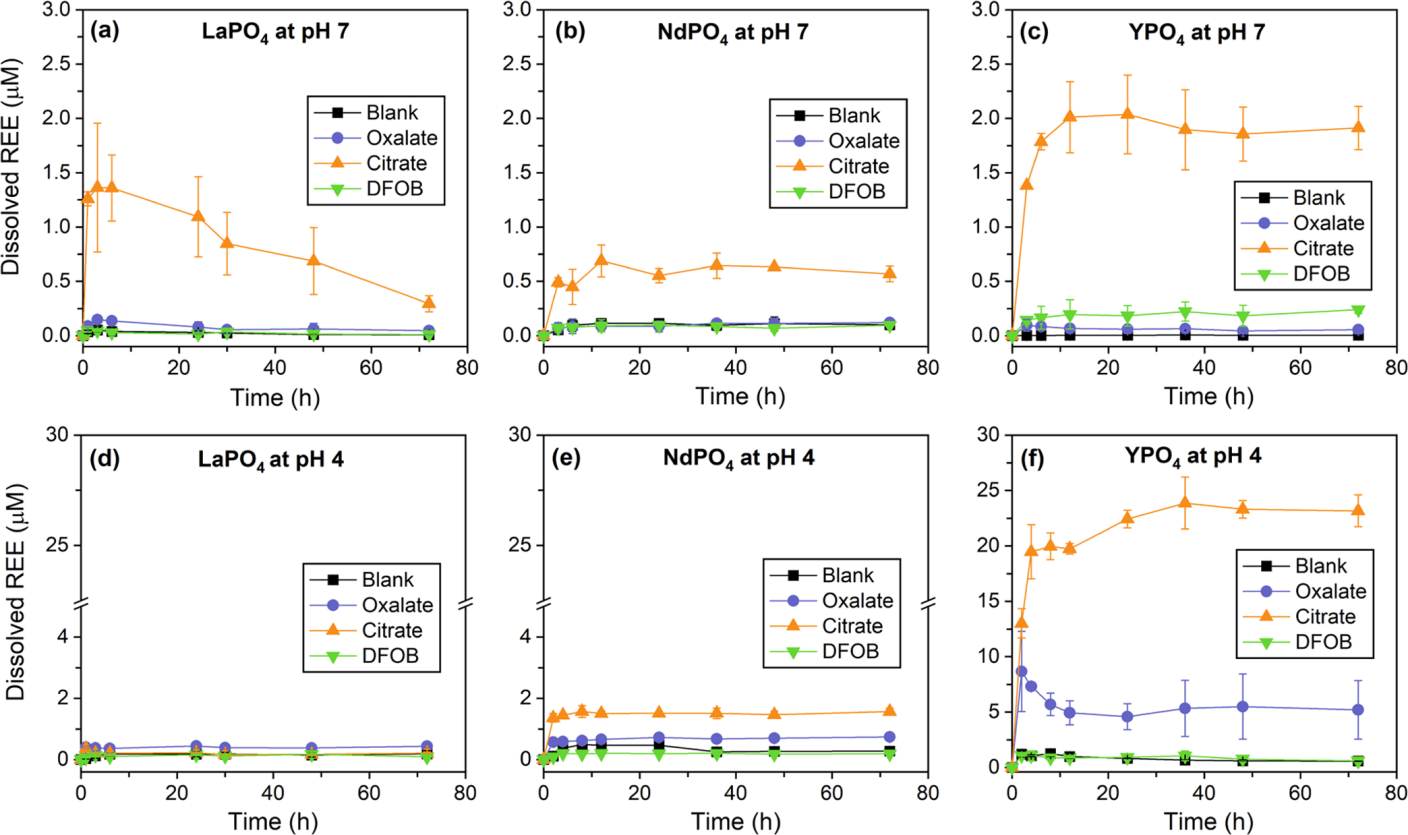
Time profiles of dissolved REE from LaPO_4_, NdPO_4_, and YPO_4_ at (a–c) pH 7 and (d–f) pH 4 in blank control, with 1 mM oxalate, 1 mM citrate, and 0.1 mM DFOB (note the breaks on the *y*-axis). Initial REE dissolution rates were obtained by fitting the data points during the beginning of reactions (see Figure 4 and Table S3). REE concentrations at the end of experiments were compared with PHREEQC modeling results (see Figure 7). To compare REE behavior difference in the presence of the same ligand, see Figure S6.

**Figure 4:**
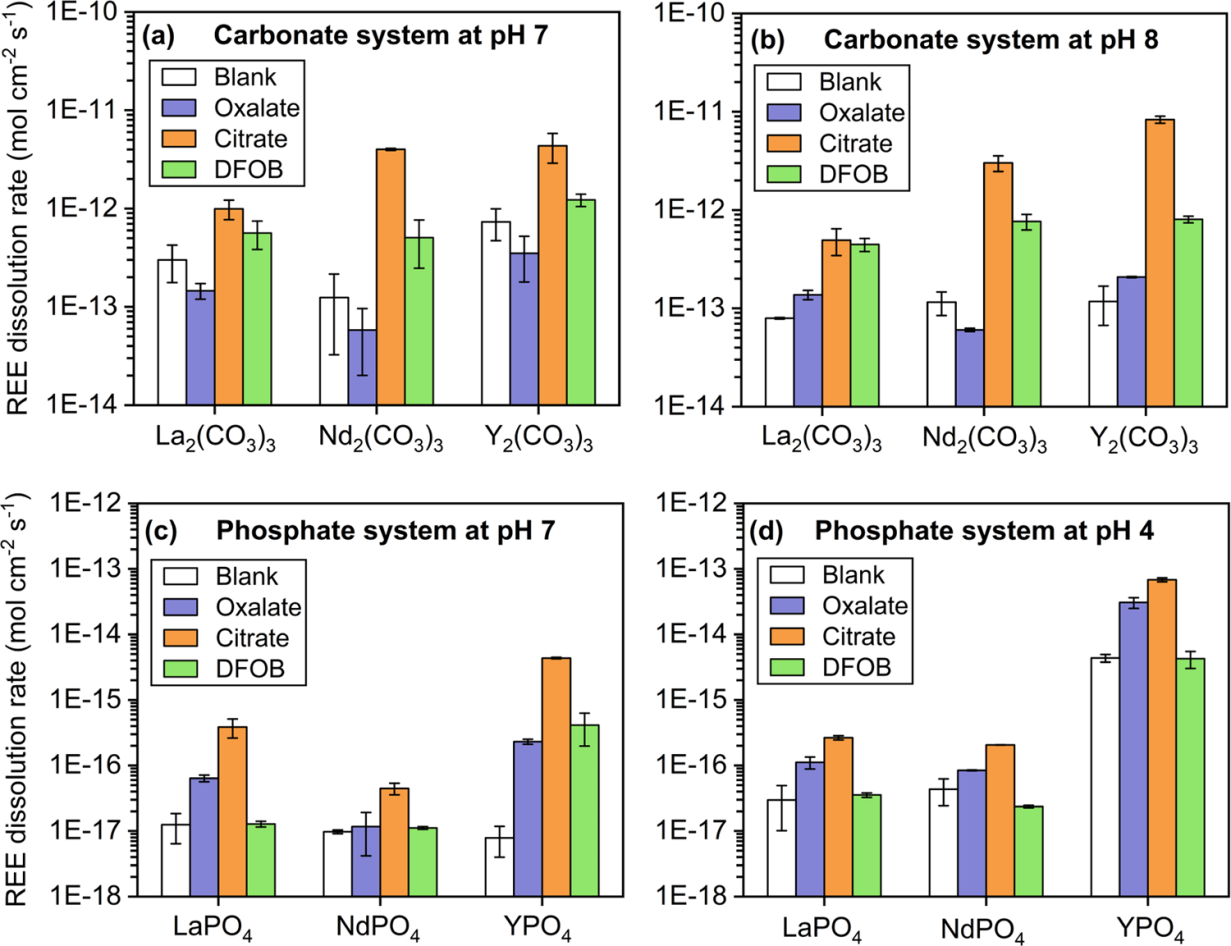
Initial REE dissolution rates (mol cm^–2^ s^–1^) in blank control, with 1 mM oxalate, 1 mM citrate, and 0.1 mM DFOB. REE dissolution rates from REE carbonate minerals at (a) pH 7 and (b) pH 8; REE dissolution rates from REE phosphate minerals at (c) pH 7 and (d) pH 4.

In the absence of organic ligands, REE dissolution was minimal for all minerals under the studied experimental conditions (Figures 2 and 3). The normalized REE dissolution rates of carbonates were approximately on the order of 10^–13^ mol cm^–2^ s^–1^ (Figure 4 and Table S3). REE dissolution rates of phosphates were much lower (∼10^–18^–10^–15^ mol cm^–2^ s^–1^), which is not surprising due to the generally lower dissolution kinetics of REE phosphates than carbonates. The surface area-normalized REE dissolution rates from phosphate minerals were consistent with previously reported values for monazite under similar conditions (∼pH 3–6).^65^

### 3.3 REE Dissolution in the Presence of Citrate

Our review on previous literature points out three major barriers for obtaining a comprehensive understanding of REE–citrate complexation chemistry. First, no consensus has been reached on the major REE-citrate species (Table S2). For example, Ohyoshi et al. suggested the predominant formation of REE(C_6_H_5_O_7_)^0^, REE(C_6_H_5_O_7_)_2_^3–^, REE(C_6_H_6_O_7_)^+^, and REE(C_6_H_6_O_7_)_2_^–^, where REE^3+^ represents a trivalent REE cation.^56^ Heller et al., on the other hand, believed that REE(C_6_H_5_O_7_)^0^, REE(C_6_H_5_O_7_)_2_^3–^, REE(C_6_H_6_O_7_)(C_6_H_5_O_7_)^2–^, and REE(C_6_H_4_O_7_)_2_^5–^ are the dominant species.^62^ Second, the calculated β^0^ of REE–citrate complexes varied notably among different studies (e.g., 7.5 vs 9.8 for EuHCit^0^; 11.4 vs 13.3 for Eu(Cit)_2_^3–^).^62,66^ Third, very few studies covered the whole REE series (Table S2). Ohyoshi et al. reported the stability constants associated with Ce, Tb, Tm, and Lu,^56^ while only Eu was studied by Heller et al.^62^

**Figure 5:**
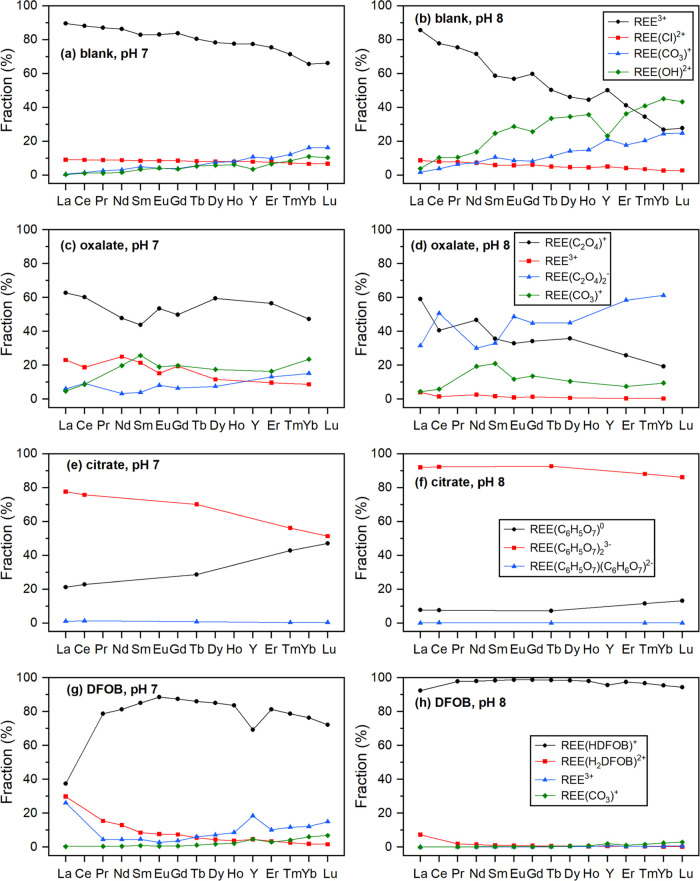
Fractions of dissolved REE species calculated using PHREEQC for REE carbonate minerals at pH 7 and pH 8: (a, b) blank control, (c, d) with 1 mM oxalate, (e, f) with 1 mM citrate, and (g, h) with 0.1 mM DFOB. REE–ligand interactions considered here are listed in Table S3. Certain data points are missing due to the lack of *K*^0^ and *K*_sp_^0^ values from the literature.

**Figure 6:**
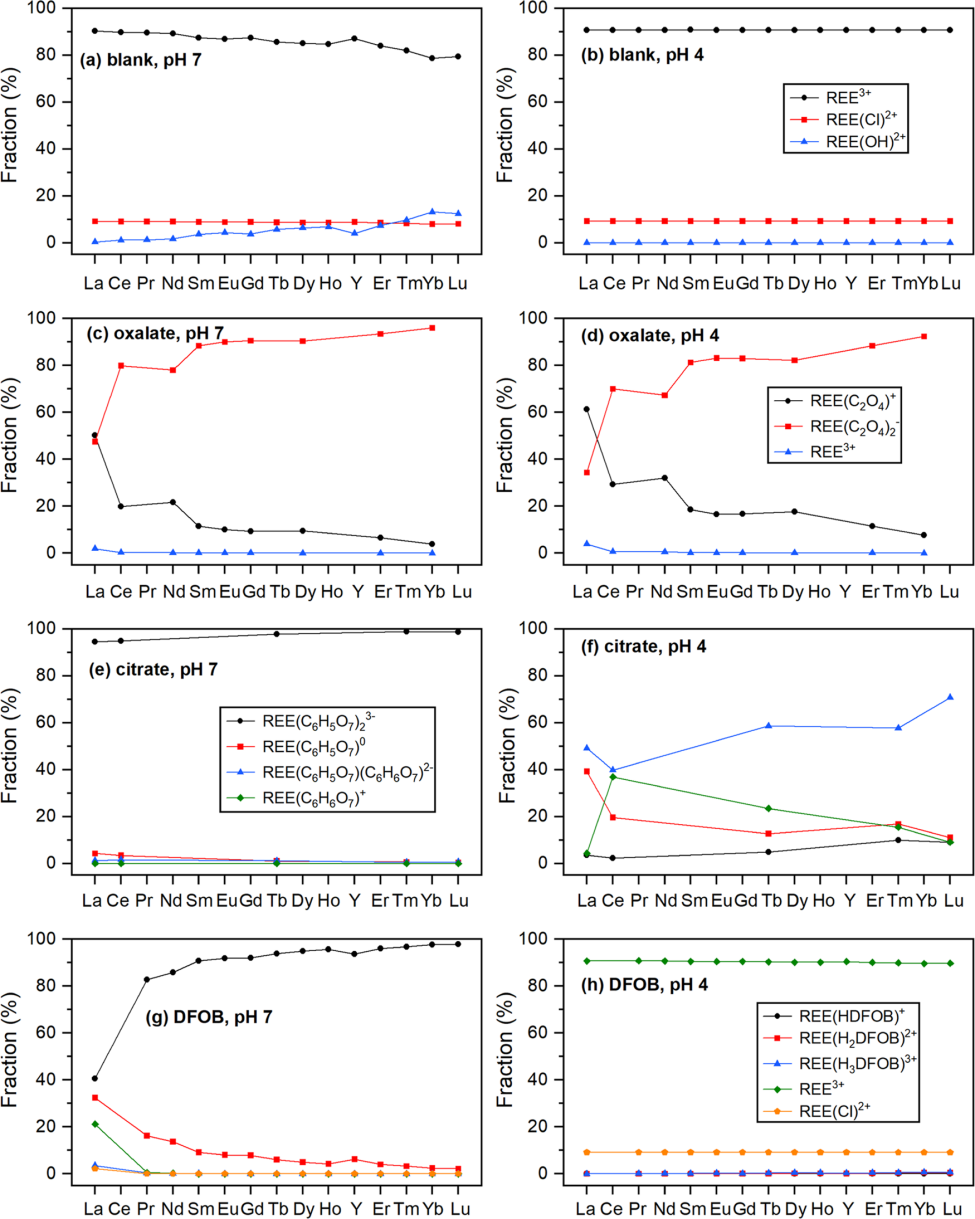
Fractions of dissolved REE species calculated using PHREEQC for REE phosphate minerals at pH 7 and pH 4: (a, b) blank control, (c, d) with 1 mM oxalate, (e, f) with 1 mM citrate, and (g, h) with 0.1 mM DFOB. REE–ligand interactions considered here are listed in Table S3. Certain data points are missing due to the lack of *K*^0^ and *K*_sp_^0^ values from the literature.

Here, we assumed REE(C_6_H_5_O_7_)^0^, REE(C_6_H_5_O_7_)_2_^3–^, REE(C_6_H_6_O_7_)^+^, and REE(C_6_H_6_O_7_)(C_6_H_5_O_7_)^2–^, the dominant REE–citrate complexes proposed by most of the previous studies, as the four major REE–citrate complexes in this study (Table S2). Thermodynamic data of REE(C_6_H_5_O_7_)^0^, REE(C_6_H_5_O_7_)_2_^3–^, and REE(C_6_H_6_O_7_)^+^ from Ohyoshi et al. and those of REE(C_6_H_6_O_7_)(C_6_H_5_O_7_)^2–^ from Hubert et al. were selected for PHREEQC calculation because these two studies had a wider coverage of REEs than other studies, and the reported species of REE–citrate complexes were complementary to each other (Table S2). To visualize the discrepancy in literature data,^56−58,62,63,66−69^ we plotted the PHREEQC modeling results for citrate-mediated REE dissolution based on reported REE-citrate speciation and corrected β^0^ from each of the previous study (colored symbols) in Figure S4. Another set of results calculated by compiling data from Ohyoshi et al. and Hubert et al. were also plotted together (black line), which represented the database used in this study. The variance of predicted REE concentrations at equilibrium in the presence of 1 mM citrate was quite notable, especially for HREEs (e.g., Yb and Lu). It can be clearly observed that for the carbonate system (at pHs 7 and 8) and the phosphate system at pH 7, the predicted dissolution profiles of REE closely resembled the calculated results from Ohyoshi et al. This is because the dominant species of REE–citrate complexes under these conditions are REE(C_6_H_5_O_7_)_2_^3–^ (e.g., ∼90% for carbonate at pH 8 and >95% for phosphate at pH 7) and REE(C_6_H_5_O_7_) (e.g., ∼20–50% for carbonate at pH 7) (Figures 5 and 6), which agreed well with what Ohyoshi et al. reported.^56^ On the other hand, the predicted profile from phosphate minerals at pH 4 corroborated with the results calculated from Hubert et al., which was expected since REE(C_6_H_5_O_7_)(C_6_H_6_O_7_)^2–^ accounted for ∼40–70% of total REE–citrate complexes under this condition (Figure 6f).

Our results show that the presence of citrate significantly promoted REE dissolution, as indicated by the highest dissolved REE concentrations at the steady state (∼5–1000 times that of blank control) and dissolution rates among all conditions (Figures 2 and 3). In the carbonate system, the REE dissolution rate with citrate is on the order of 10^–13^–10^–12^ mol cm^–2^ s^–1^, while it varies greatly for REE phosphate minerals, ranging from 10^–17^ to 10^–14^ mol cm^–2^ s^–1^ (Figure 4). For both systems, REE dissolution rates in the presence of citrate are at least 1 and up to 3 orders of magnitude higher than the blank control and oxalate. PHREEQC calculations suggested that such enhancements are due to the formation of soluble and stable REE–citrate complexes (Figures 5 and 6). The main REE species in the carbonate systems were REE(C_6_H_5_O_7_)_2_^3–^ (β^0^ = 12.9–14.9) at both pH 7 (∼50–80%) and pH 8 (∼90%) (Figure 5). In the phosphate system, REE(C_6_H_5_O_7_)_2_^3–^ (>95%) and REE(C_6_H_5_O_7_)(C_6_H_6_O_7_)^2–^ (β^0^ = 12.2–14.0) (∼40–70%) were dominant at pHs 7 and 4, respectively (Figure 6).

### 3.4 REE Dissolution in the Presence of Oxalate

Intriguingly, the addition of oxalate either inhibited or promoted REE dissolution compared to the blank control under the experimental conditions (Figures 2 and 3). For example, oxalate markedly promoted Y dissolution from YPO_4_ (pH 4 and 7) and La dissolution from La_2_(CO_3_)_3_ (pH 8), whereas it slightly inhibited the dissolution of La, Nd, and Y from carbonate minerals (pH 7). The divergent effect of oxalate was particularly evident for YPO_4_ at pH 4, in which Y was quickly released during the first 3 h (formation of Y–oxalate complexes), followed by a sharp decline during 3–24 h until reaching a steady state (precipitation of Y-oxalate) (Figure 3f). Similar results were also observed for Y_2_(CO_3_)_3_ at pH 7 and LaPO_4_ at pH 7 (Figures 2c and 3a), which can also be clearly visualized from the normalized REE dissolution rates in Figure 4. Such a phenomenon is likely due to two competitive processes: (1) release of REEs from minerals via the formation of REE–oxalate complexes, with REE(C_2_O_4_)^+^ and REE(C_2_O_4_)_2_^–^ being the dominant species and (2) precipitation of the REE-oxalate solid phase. PHREEQC calculations revealed that in the carbonate system, REE(C_2_O_4_)^+^ accounted for ∼50–60% of total dissolved REE species at pH 7, whereas at pH 8 REE(C_2_O_4_)^+^ and REE(C_2_O_4_)_2_^–^ accounted for ∼20–60 and ∼30–60% of dissolved REE, respectively (Figure 5 and Table S4). For the phosphate system, REE(C_2_O_4_)^+^ and REE(C_2_O_4_)_2_^–^ constituted over 95% of dissolved REEs at both pHs 7 and 4 (Figure 6 and Table S4).

Furthermore, the saturation indexes (SIs) of REE–oxalate complexes were positive for both the carbonate (∼3.7–6.6) and phosphate systems (∼4.3–6.5), indicating that precipitation of REE–oxalate complexes was thermodynamically favorable under these conditions (Table S5). Note that the lower SI of REE-oxalates at a higher pH can be ascribed to the more favored precipitation of REE-hydroxides, which is supported by their increase in SI. For example, as the pH rises from 7 to 8 in the carbonate system, the SI of La-oxalate decreases from 5.76 to 5.48, while that of La-hydroxide increases from −3.81 to −1.99. In addition, although REE–oxalate complexes in the phosphate system remain insoluble at both pHs 7 and 4, a noticeable increase in SI from pH 7 to 4 can be observed (Table S5). Such an increase is especially notable for Y, whose SI rises from 3.07 to 4.27 from pH 7 to 4, which could be responsible for the sharp decrease in dissolved Y concentration after initial release from YPO_4_ at pH 4 (Figure 3f). We further calculated the SI of REE–oxalate complexes in the phosphate system at pH 1 and found that the SI was significantly decreased to near-zero or negative (e.g., 0.76 for Nd-oxalate and −1.26 for Y-oxalate), suggesting that the fate of REE-oxalates (i.e., its existence as dissolved or precipitated species) is strongly influenced by the pH (Table S5). Therefore, the overall effect of oxalate varies depending on the β^0^ and SI of REE–oxalate complexes and is also highly kinetically controlled.

### 3.5 REE Dissolution in the Presence of DFOB

DFOB notably promoted REE dissolution from carbonate minerals at both pHs 7 and 8 (Figure 2). The dissolved Y concentration from Y_2_(CO_3_)_3_ at pH 7 reached ∼150 μM in 2 h with DFOB (Figure 2c). The dissolution rate of Nd_2_(CO_3_)_3_ at pH 8 in the presence of DFOB (7.65 ± 1.38 × 10^–13^ mol cm^–2^ s^–1^) was almost 7-fold that of the blank control (1.16 ± 0.31 × 10–13 mol cm^–2^ s^–1^) (Table S3). PHREEQC calculations confirmed the formation of ReHDFOB^+^ complex as the dominant REE species, which accounted for ∼40–90 and >90% of dissolved REEs at pHs 7 and 8, respectively (Figure 5).

On the other hand, dissolution of REE phosphate minerals was largely unaffected by DFOB at both pHs 7 and 4 compared to the control blank (Figures 3 and 4), which might be partly due to the lower concentration of DFOB (0.1 mM) compared to citrate and oxalate (1 mM). It can be seen from Figure S2 and Table 1 that as the pH increases, DFOB forms more stable complexes with REE (e.g., log β^0^ of REE(H_3_DFOB)^3+^ = 4.88–6.53; log β^0^ of REE(H_2_DFOB)^2+^ = 8.45–12.02; log β^0^ of REE(HDFOB)^+^ = 11.59–16.69). This corroborated with the PHREEQC modeling results that ∼90% of dissolved REEs exists as free cations at pH 4, whereas REE–DFOB complexes constitute ∼80–100% of dissolved REEs at pH 7 (Figure 6).

Overall, 1 mM citrate dramatically enhanced the dissolution of REEs from both REE carbonate and phosphate minerals by forming stable and soluble REE complexes. The overall effect of 1 mM oxalate varied due to the formation of REE–oxalate complexes and the precipitation of REE oxalate solid phases. The presence of 0.1 mM DFOB displayed a mildly promoting effect on dissolution of REE carbonates but a minor effect on REE phosphates.

### 3.6 Organic Ligand-Mediated REE Fractionation

**Figure 7:**
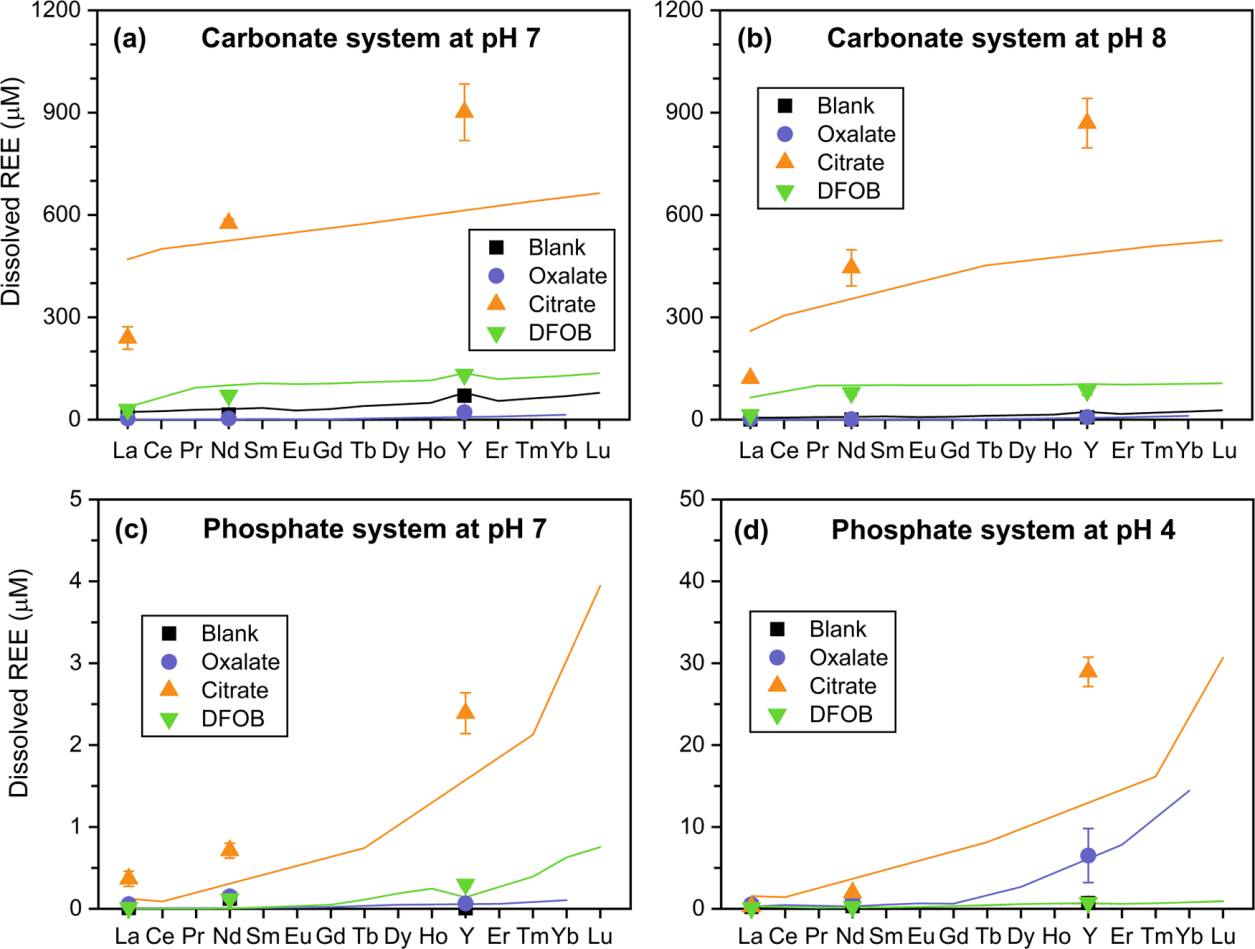
Comparison between experimental data and PHREEQC modeling results on dissolved REE concentrations at equilibrium (experimental data of La, Nd, and Y are plotted as symbols; PHREEQC modeling results for all REEs are plotted as lines). Experiments were conducted in blank control (in black), with 1 mM oxalate (in purple), 1 mM citrate (in orange), and 0.1 mM DFOB (in green). PHREEQC modeling results were based on published thermodynamic data.

The dissolution profiles of different REE are shown in Figures S5 and S6. It can be seen that all three ligands preferentially dissolved Y over La and Nd from the minerals, suggesting a trend toward the fractionation of HREEs in solution. To better visualize the fractionation trend and unveil underlying mechanisms, steady-state REE concentrations were plotted (as symbols) to compare with predicted results by PHREEQC thermodynamic calculations (connected lines) in Figure 7. Y was placed between Ho and Er because of their similar ionic radii. Note that due to incomplete literature data as previously discussed (Table 1), the connected lines in Figure 7 may not accurately depict the behaviors of every REE.

The dissolution profiles of selected REEs by organic ligands featured a distinct fractionation toward HREE enrichment in solution, and the dissolved REE concentration at equilibrium followed the order of Y > Nd > La (Figure 7). The dissolved Y concentration at equilibrium from Y_2_(CO_3_)_3_ at pH 7 reached ∼900 μM, which was significantly higher than those of Nd (∼550 μM) and La (∼240 μM) (Figure 7a). Similarly, the equilibrium concentration of Y in the phosphate system at pH 4 (∼23 μM) was more than 10 and 100 times higher than those of Nd (∼1.6 μM) and La (∼0.2 μM), respectively (Figure 7d). Even though oxalate and DFOB exhibited varied impacts on REE dissolution under different conditions, similar patterns were observed in general. Such a trend was also consistent with PHREEQC modeling results that the predicted REE concentration at equilibrium steadily increases as the atomic number of REE increases (from La to Lu) under all conditions. Our experimental data matched well with the trend lines predicted by PHREEQC, especially for oxalate and DFOB (Figure 7). As discussed earlier, the precipitation of REE-oxalate could be responsible for the minor increase in the equilibrium REE concentration from La to Lu. Interestingly, if the precipitation of REE-oxalate was not considered in PHREEQC, the predicted REE concentrations also displayed an obvious increasing trend from La to Lu, which indicates the higher affinity of oxalate toward HREE. We also point out that despite the discrepancy between experimental data and modeling results for citrate, which are likely due to incomplete thermodynamic data of REE–citrate complexation from the literature, the overall pattern still illustrated an evident fractionation trend toward HREE enrichment.

Such a fractionation trend can be mainly attributed to the difference in metal–ligand complex stability. According to Pearson’s hard/soft acid/base (HSAB) principle, hard Lewis acids and hard Lewis bases form thermodynamically more stable complexes, and vice versa for soft acid–soft base complexes.^70^ Hard acids and bases are typically characterized by a high charge density, small ionic radius, and low polarizability. By this definition, all trivalent REEs are hard acids, and all three organic ligands are hard bases. In addition, REE feature a continuous and steady decrease in ionic radii as their atomic number increases (lanthanide contraction), and thus HREEs are harder acids than LREEs. Although comparing the hardness of certain organic ligands can be speculative, Xu et al. calculated the index of chemical hardness for common organic ligands, which indicated oxalate as a softer base than citrate.^71^ Although such data is not available for DFOB, we can reasonably postulate that DFOB is also a softer base than citrate considering that the carboxylate groups in citrate might be less polarizable than hydroxamate groups in DFOB. Hence, these hard-base ligands, especially citrate, formed more stable complexes with HREEs and enhanced both the rate and extent of dissolution compared to LREEs. This explanation can be further substantiated by results from a recent study which showed that the complexation of REEs by ethylenediaminetetraacetate (EDTA), a soft base ligand, led to an opposite fractionation pattern toward the enrichment of LREE in solution.^72^ Besides complex stability, the higher *K*_sp_^0^ of HREE minerals than that of LREE minerals could also facilitate HREE dissolution (Table 1).

## 4 Environmental Implications

This work illustrates the strong influence of organic ligands on REE mobilization and fractionation under environmentally relevant conditions. Citrate substantially enhanced the rate and extent of REE dissolution from host minerals, while DFOB showed a mild promotion effect, and oxalate showed both an enhancing and an inhibiting effect depending on the environmental conditions. The surface area-normalized dissolution rates of La, Nd, and Y in the presence of organic ligands provide a reference baseline for future research. The PHREEQC modeling results matched well with experimental data and confirmed the formation of REE–ligand complexes under different conditions. Organic ligands used in this study showed a fractionation pattern that facilitated HREE dissolution over LREEs. Our results suggest that the stability of different REE–ligand complexes and the solubility of relevant mineral phases under the studied pH might be the primary driving force behind the REE fractionation pattern and the varied effects of organic ligands.

Understanding the kinetics and thermodynamics of REE dissolution from their primary host minerals in the presence of organic ligands has profound implications for investigating the geochemical behaviors of REE. We highlight that investigation of REE behaviors under simulated but simplified natural conditions can contribute to a better prediction of REE mobilization and transport in more complex natural environments. The ubiquitous presence of natural organic matter (NOM) (e.g., humic and fulvic substances) also heavily influence REE mobilization, especially at solid–liquid interfaces in surface waters and wetlands.^73^ While research in this direction is still difficult, owing to the chemical complexity and variability of NOM, our results using simple model organic ligands can provide insights for future studies on REE–NOM interactions. We also note that although HREEs form thermodynamically more stable complexes with NOM compared to LREEs, LREE enrichment was observed in surface and groundwaters of many countries (e.g., U.S., France, Brazil, etc.).^74−76^ These findings suggest that other biogeochemical reactions are also involved in REE transport and redistribution after dissolution from host phases, such as the formation of or incorporation into secondary minerals, coprecipitation with colloids and suspended matter, redox reactions (e.g., Ce^3+^/Ce^4+^ and Eu^2+^/Eu^3+^), or microbial uptake.^25^ Elucidating the REE behavior in these complex systems in the presence of organic ligands warrants future research.

A deeper knowledge of REE–ligand interactions and impacts of pH on REE dissolution and complexation can also shed light on a more mature design of REE extraction. Although interest in using organic ligands (e.g., citrate) in REE extraction has been steadily growing,^8−10^ the lack of a systematic understanding of REE–citrate interactions restricts further progress in this direction. The underlying kinetics and thermodynamics of REE–citrate complexation revealed by our results can contribute to the applications of organic ligands in real practice. Important features of organic ligands, including ligand-promoted dissolution and fractionation of REE as well as pH-dependent behaviors as demonstrated by our findings, can be exploited to design chemical-/energy-efficient methods for REE separation/purification and to overcome the limitations of traditional biphasic solvent extraction.^77^ For example, oxalate can form a soluble REE complex at low pHs and then precipitate as the pH increases, which can be employed to separate REEs from the aqueous phase and even differentiate LREEs/HREEs by adjusting the solution chemistry, given its fractionation tendency toward HREEs. In addition, this study helps fill up the current knowledge gap in the kinetic/thermodynamic database and modeling of metal–ligand complexation. Advances in computational methods in this regard can enable rapid screening of suitable ligands and elaborate design of treatment processes for REE extraction/separation without solely relying on labor-intensive experimental works.

## Supporting Information Available

Details on chemicals and analytical methods; summary of experimental design; summary of literature reported REE–citrate complexation chemistry; normalized dissolution rates of REE minerals in the presence of organic ligands; predicted major REE–ligand species under experimental settings; saturation index of REE–oxalate and REE–hydroxide complexes at different pHs; dissolution profiles of REE minerals as a function of pH; speciation of oxalate, citrate, and DFOB over pH; SEM images of REE minerals; modeling results of REE-citrate species from literature, and REE dissolution profiles for carbonate and phosphate minerals (PDF)
