# Supplementary material for: Organic Ligand-Mediated Dissolution and Fractionation of Rare-Earth Elements (REEs) from Carbonate and Phosphate Minerals
Source: ACS Earth Space Chem. 2024 Apr 25;8(5):1048–61. doi: 10.1021/acsearthspacechem.4c00009 (PMC11103772; doi:10.1021/acsearthspacechem.4c00009)
Supplement: Supplementary file 1 — sp4c00009_si_001.pdf [file sp4c00009_si_001.pdf]

Supporting Information

Organic ligand mediated dissolution and fractionation of rare earth elements (REE) from carbonate and phosphate minerals

Yinghao Wen†, Pan Liu†, Qian Wang, Simin Zhao, Yuanzhi Tang*

School of Earth and Atmospheric Sciences, Georgia Institute of Technology, 311 Ferst Drive, Atlanta, Georgia 30332, United States

*Corresponding author:

Yuanzhi Tang

Email: yuanzhi.tang@eas.gatech.edu

Phone: 404-894-3814

Total 14 pages

2 texts

5 tables

6 figures

Text S1. Chemicals and reagents

All REE minerals and chemicals used are >99.9% purity (trace-metal grade). La(III) carbonate, La(III), Nd(III), and Y(III) chlorides, and the methanesulfonate (mesylate) salt of DFOB $[(C_{25}H_{46}N_5O_8NH_3)^+(CH_3SO_3)^-]$ were purchased from Sigma Aldrich (St. Louis, USA). Nd(III) and Y(III) carbonates were purchased from Alfa Aesar (Haverhill, USA). Disodium oxalate ($Na_2C_2O_4$) was purchased from J.T. Baker (Philipsburg, USA). Trisodium citrate ($Na_3C_6H_5O_7$) was purchased from Amresco (Boise, USA). Indium internal standard (TraceCERT) and REE mix standard (TraceCERT) were purchased from Sigma-Aldrich. Ultrapure deionized water (18.2 MΩ·cm) produced by a Barnstead Nanopure system (Thermo Fisher Scientific, Waltham, USA) was used in all solutions.

Text S2. Analytical methods

X-ray diffraction (XRD). The mineralogy of REE minerals was examined using a Panalytical Empyrean multipurpose diffractometer (Cu Kα). XRD patterns were recorded at 45 kV and 40 mA over the range of 10−50 °2θ with a step size of 0.03 °2θ and a contact time of 15 s/step using a PIXcel 3D-Medipi×3 1×1 detector. The obtained XRD patterns were analyzed using JADE with the ICDD database.

Scanning electron microscopy (SEM). The morphology of REE minerals was examined using a Hitachi SU8230 SEM. Samples were dusted on sample stages with carbon tapes, and SEM images were taken at 5 kV and 10 μA with a working distance of 5 mm.

Brunauer-Emmett-Teller (BET) surface area. In order to normalize the dissolution rates of REE from minerals, the BET specific surface area of pristine REE minerals was measured by He adsorption at liquid He temperature using a Micromeritics TriStar 3000 BET surface area analyzer (Norcross, USA). Approximately 0.2 g sample was degassed at 150 °C for 3 h under vacuum prior to the He adsorption measurement.

Inductively coupled plasma mass spectrometry (ICP-MS). REE concentrations in collected aliquots were measured using an Agilent 7500a ICP-MS. All aliquots were acidified with 2% HNO_3 (v/v) and spiked with 10 ppb of indium (In) as an internal standard. A series of calibration standards (0−200 ppb REE) was prepared using REE mix standard, and each standard solution was spiked with 10 ppb of In as well. The mass spectrometer was carefully tuned using tuning solution containing 10 ppb of Li, Y, Ce, Tl, and Co. The presence of oxide (MO^+/M^+) and double-charged ions (M^{2+}/M^+) was tuned to below 1% and 2%, respectively. Counts of ^{72}Y, ^{139}La, ^{143}Nd, ^{145}Nd, and ^{146}Nd were measured and normalized by ^{115}In to correct instrument drift. Concentration of Y, La, and Nd was calculated based on linear regressions of the REE standards. REE calibration standards were re-measured every 20 samples to ensure accuracy.

Table S1. Summary of REE carbonate and phosphate minerals and experiment design in this study. REE-phosphate minerals were synthesized following procedures reported by Lucas et al. (2004).[1]

Mineral	Source or preparation method	Major mineral phase by XRD	BET surface area (m^2/g)	Experiment conditions
$La_2(CO_3)_3$	La(III) carbonate (Sigma Aldrich)	Lanthanite-(La): [$La_2(CO_3)_3 \cdot 8H_2O$]	1.95 ± 0.04	**Ligand**: Blank, 1 mM oxalate, 1 mM citrate, or 0.1 mM DFOB<br>**pH**: 7.0 and 8.0<br>**Reaction time**: 0−48 h
$Nd_2(CO_3)_3$	Nd(III) carbonate (Alfa Aesar)	Tengerite-(Nd): [$Nd_2(CO_3)_3 \cdot 2–3H_2O$]	5.40 ± 0.02	
$Y_2(CO_3)_3$	Y(III) carbonate (Alfa Aesar)	Tengerite-(Y): [$Y_2(CO_3)_3 \cdot 2–3H_2O$]	6.60 ± 0.01	
$LaPO_4$	Synthesized by precipitation of $LaCl_3 \cdot 7H_2O$ and H_3PO_4 with a molar ratio of 1:10	Rhabdophane-(La): [$LaPO_4 \cdot H_2O$]	76.71 ± 0.31	**Ligand**: Blank, 1 mM oxalate, 1 mM citrate, or 0.1 mM DFOB<br>**pH**: 4.0 and 7.0<br>**Reaction time**: 0−72 h
$NdPO_4$	Synthesized by precipitation of $NdCl_3 \cdot 6H_2O$ and H_3PO_4 with a molar ratio of 1:10	Rhabdophane-(Nd): [$NdPO_4 \cdot H_2O$]	115.20 ± 0.25	
YPO_4	Synthesized by precipitation of $YCl_3 \cdot 6H_2O$ and H_3PO_4 with a molar ratio of 1:10	Churchite-(Y): [$YPO_4 \cdot 2H_2O$]	4.22 ± 0.02	

Table S2. Summary of previous studies on REE-citrate complexation chemistry. REE^{3+} represents trivalent REE cation. $REE(C_6H_5O_7)^0$, $REE(C_6H_5O_7)_2^{3-}$, $REE(C_6H_6O_7)^+$, and $REE(C_6H_6O_7)(C_6H_5O_7)^{2-}$ (highlighted in bold) were considered as important REE-citrate complexes in this study.

Method	Experimental conditions	Proposed REE-citrate complexes	REE studied	Reference
Spectrophotometry	1 M NaCl, $1.3 \le pH \le 2.7$	**$REE(C_6H_5O_7)^0$, $REE(C_6H_6O_7)^+$**, $REE(C_6H_7O_7)^{2+}$	La	ref[2]
Ion exchange	0.1 M NaCl, $2.0 \le pH \le 3.2$	**$REE(C_6H_5O_7)^0$, $REE(C_6H_6O_7)^+$, $REE(C_6H_5O_7)_2^{3-}$**, $REE(C_6H_6O_7)_2^-$	Ce, Pm, Tb, Tm, Lu	ref[3]
Solvent extraction	0.15 M $LiClO_4$, $3.5 < pH < 5.5$	**$REE(C_6H_5O_7)(C_6H_6O_7)^{2-}$**	La, Pr, Nd, Pm, Sm, Eu, Gd, Tb, Dy, Ho, Er, Tm, Tb, Lu	ref[4]
Potentiometry	0.1 M $LiClO_4$, $1 < pH < 13$	**$REE(C_6H_5O_7)^0$, $REE(C_6H_6O_7)^+$**, $REE(C_6H_7O_7)^{2+}$, **$REE(C_6H_5O_7)_2^{3-}$, $REE(C_6H_5O_7)(C_6H_6O_7)^{2-}$**, $REE(OH)(C_6H_5O_7)^-$, $REE_3(OH)_4(C_6H_5O_7)_4^{7-}$	Nd	ref[5]
/	/	**$REE(C_6H_5O_7)^0$**	La, Ce, Pr, Nd, Sm, Eu, Gd, Tb, Dy, Ho, Y, Er, Tm, Tb, Lu	ref[6]
/	/	**$REE(C_6H_5O_7)^0$, $REE(C_6H_5O_7)_2^{3-}$**	Lu	ref[7]
Solvent extraction	6.6 M $NaClO_4$, pH 3.6	**$REE(C_6H_5O_7)^0$, $REE(C_6H_5O_7)_2^{3-}$**	Eu	ref[8]
Solvent extraction	0.1−6.6 M $NaClO_4$, pH 3.6	**$REE(C_6H_5O_7)^0$, $REE(C_6H_5O_7)_2^{3-}$**	Eu	ref[9]
Spectrophotometry	0.1 M $NaClO_4$, $2 < pH < 13$	**$REE(C_6H_5O_7)^0$, $REE(C_6H_5O_7)_2^{3-}$, $REE(C_6H_5O_7)(C_6H_6O_7)^{2-}$**, $REE(C_6H_4O_7)_2^{5-}$	Eu	ref[10]
Potentiometry	1.06 M $NaClO_4$, $2 < pH < 5$	**$REE(C_6H_5O_7)^0$, $REE(C_6H_6O_7)^+$, $REE(C_6H_5O_7)_2^{3-}$, $REE(C_6H_5O_7)(C_6H_6O_7)^{2-}$**	Nd	ref[11]

Table S3. Normalized dissolution rates of REE minerals in the presence of organic ligands calculated in this study (0.1 M NaCl was present in all treatment groups).

Minerals	pH	Organic ligand	Normalized REE dissolution rate ($mol·cm^{-2}s^{-1}$)
$La_2(CO_3)_3$	7	/	$3.00 \pm 1.24 \times 10^{-13}$
		1 mM oxalate	$1.46 \pm 0.26 \times 10^{-13}$
		1 mM citrate	$9.93 \pm 2.23 \times 10^{-13}$
		0.1 mM DFOB	$5.64 \pm 1.82 \times 10^{-13}$
	8	/	$7.32 \pm 2.61 \times 10^{-14}$
		1 mM oxalate	$1.37 \pm 0.15 \times 10^{-13}$
		1 mM citrate	$4.94 \pm 1.50 \times 10^{-13}$
		0.1 mM DFOB	$4.46 \pm 0.68 \times 10^{-13}$
$Nd_2(CO_3)_3$	7	/	$1.24 \pm 0.92 \times 10^{-13}$
		1 mM oxalate	$5.80 \pm 3.79 \times 10^{-14}$
		1 mM citrate	$4.00 \pm 0.10 \times 10^{-12}$
		0.1 mM DFOB	$5.06 \pm 2.59 \times 10^{-13}$
	8	/	$1.16 \pm 0.31 \times 10^{-13}$
		1 mM oxalate	$6.04 \pm 0.23 \times 10^{-14}$
		1 mM citrate	$3.02 \pm 0.55 \times 10^{-12}$
		0.1 mM DFOB	$7.65 \pm 1.38 \times 10^{-13}$
$Y_2(CO_3)_3$	7	/	$7.32 \pm 2.61 \times 10^{-13}$
		1 mM oxalate	$3.51 \pm 1.72 \times 10^{-13}$
		1 mM citrate	$4.35 \pm 1.45 \times 10^{-12}$
		0.1 mM DFOB	$1.22 \pm 0.17 \times 10^{-12}$
	8	/	$1.17 \pm 0.50 \times 10^{-13}$
		1 mM oxalate	$2.08 \pm 0.03 \times 10^{-13}$
		1 mM citrate	$8.33 \pm 0.68 \times 10^{-12}$
		0.1 mM DFOB	$8.04 \pm 0.63 \times 10^{-13}$
$LaPO_4$	7	/	$1.25 \pm 0.61 \times 10^{-17}$
		1 mM oxalate	$6.38 \pm 0.74 \times 10^{-17}$
		1 mM citrate	$3.87 \pm 1.24 \times 10^{-16}$
		0.1 mM DFOB	$1.28 \pm 0.13 \times 10^{-17}$
	4	/	$2.97 \pm 1.96 \times 10^{-17}$
		1 mM oxalate	$1.12 \pm 0.23 \times 10^{-16}$
		1 mM citrate	$2.65 \pm 0.20 \times 10^{-16}$
		0.1 mM DFOB	$3.54 \pm 0.28 \times 10^{-17}$
$NdPO_4$	7	/	$9.71 \pm 0.61 \times 10^{-18}$
		1 mM oxalate	$1.17 \pm 0.75 \times 10^{-17}$
		1 mM citrate	$4.46 \pm 0.90 \times 10^{-17}$
		0.1 mM DFOB	$1.11 \pm 0.05 \times 10^{-17}$
	4	/	$4.34 \pm 1.90 \times 10^{-17}$
		1 mM oxalate	$8.43 \pm 0.12 \times 10^{-17}$
		1 mM citrate	$2.05 \pm 0.01 \times 10^{-16}$
		0.1 mM DFOB	$2.37 \pm 0.01 \times 10^{-17}$
YPO_4	7	/	$7.87 \pm 3.90 \times 10^{-18}$
		1 mM oxalate	$2.31 \pm 0.20 \times 10^{-16}$
		1 mM citrate	$4.35 \pm 0.12 \times 10^{-15}$
		0.1 mM DFOB	$4.13 \pm 2.16 \times 10^{-16}$
	4	/	$4.36 \pm 0.58 \times 10^{-15}$
		1 mM oxalate	$3.07 \pm 0.56 \times 10^{-14}$
		1 mM citrate	$6.84 \pm 0.47 \times 10^{-14}$
		0.1 mM DFOB	$4.26 \pm 1.23 \times 10^{-15}$

Table S4. Main REE species under different experimental settings based on PHREEQC calculations.

Ligand	REE-carbonate system		REE-phosphate system	
	pH 7	pH 8	pH 7	pH 4
Blank	REE^{3+} (~70–90%)	REE^{3+} (~30–90%)<br>$REE(OH)^{2+}$ (~5–45%)	REE^{3+} (~80–90%)	REE^{3+} (~90%)
Oxalate	$REE(C_2O_4)^+$ (~50–60%)	$REE(C_2O_4)_2^-$ (~30–60%)<br>$REE(C_2O_4)^+$ (~20–60%)	$REE(C_2O_4)_2^-$ (~50–95%)<br>$REE(C_2O_4)^+$ (~5–50%)	$REE(C_2O_4)_2^-$ (~35–90%)<br>$REE(C_2O_4)^+$ (~10–60%)
Citrate	$REE(C_6H_5O_7)_2^{3-}$ (~50–80%)<br>$REE(C_6H_5O_7)$ (~20–50%)	$REE(C_6H_5O_7)_2^{3-}$ (~90%)	$REE(C_6H_5O_7)_2^{3-}$ (~95–100%)	$REE(C_6H_5O_7)(C_6H_6O_7)^{2-}$ (~40–70%)<br>$REE(C_6H_5O_7)$ (~10–40%)<br>$REE(C_6H_6O_7)^+$ (~5–40%)
DFOB	$REE(HDFOB)^+$ (~40–90%)	$REE(HDFOB)^+$ (~90–100%)	$REE(HDFOB)^+$ (~40–100%)	REE^{3+} (~90%)

Table S5. Saturation index (SI) of REE-oxalate complexes and REE hydroxides (values in parentheses) in the presence of 1 mM oxalate and at different pH modeled by PHREEQC (SI of Pr, Tb, Ho, Tm, Lu-oxalate complexes are not available in PHREEQC). La, Nd, and Y (highlighted in bold) are the three REE experimentally studied in this work. SI at pH 1 for the phosphate system was modeled to indicate the dissolution of REE-oxalates under strongly acidic condition.

**REE**	**Carbonate system**				**Phosphate system**					
	**pH 7**		**pH 8**		**pH 7**		**pH 4**		**pH 1**	
	**REE-Oxalate**	**REE-Hydroxide**	**REE-Oxalate**	**REE-Hydroxide**	**REE-Oxalate**	**REE-Hydroxide**	**REE-Oxalate**	**REE-Hydroxide**	**REE-Oxalate**	**REE-Hydroxide**
**La**	**5.76**	**-3.81**	**5.48**	**-1.99**	**5.18**	**-2.81**	**5.35**	**-11.75**	**-0.91**	**-23.83**
Ce	6.34	-2.52	5.91	-0.77	6.23	-1.32	6.33	-10.26	0.27	-22.24
Pr		-2.13		-0.36		-0.96		-9.90		-21.74
**Nd**	**6.62**	**-0.53**	**6.47**	**1.36**	**6.32**	**0.60**	**6.47**	**-8.34**	**0.76**	**-20.14**
Sm	6.56	-0.89	6.03	0.80	6.58	0.38	6.70	-8.56	1.21	-20.25
Eu	6.50	-0.27	5.95	1.42	6.47	0.99	6.61	-7.95		
Gd	6.57	0.12	6.07	1.84	6.53	1.33	6.58	-7.61	1.23	-19.24
Tb		-0.61		1.04		0.65		-8.29		-19.82
Dy	5.40	-1.17	4.76	0.53	5.40	0.15	5.53	-8.79	0.55	-20.23
Ho		-0.50		1.13		0.79		-8.15		-19.56
**Y**	**4.22**	**-0.98**	**3.69**	**0.72**	**3.07**	**0.25**	**4.27**	**-8.69**	**-1.26**	**-20.20**
Er	4.47	-0.59	3.75	1.02	4.54	0.73	4.68	-8.21	-0.11	-19.56
Tm		-0.52		1.06		0.83		-8.11		
Yb	4.06	-0.71	3.32	1.43	4.12	0.65	4.38	-8.28	-0.14	-19.50
Lu		-0.38		1.19		0.99		-7.94	-0.91	-19.15

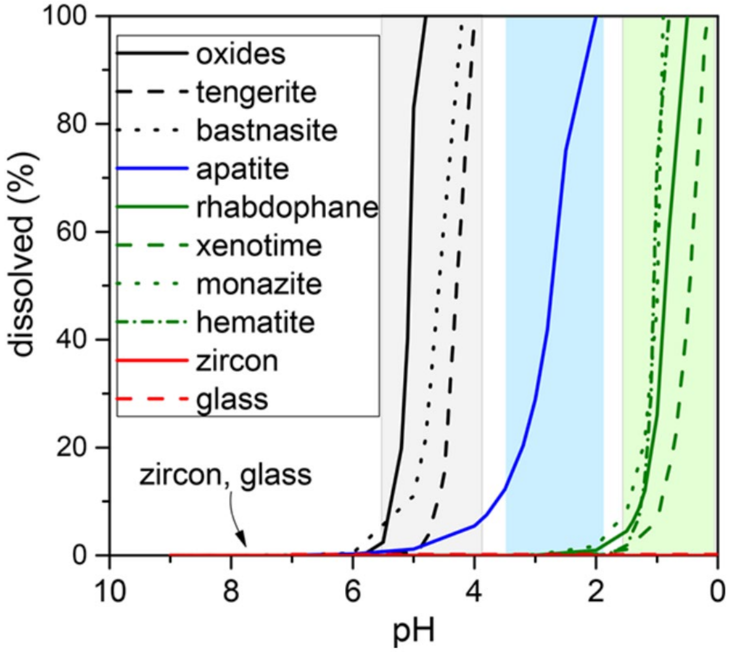

Figure S1. Dissolution profiles of different REE-bearing phases as a function of pH predicted by PHREEQC using published thermodynamic data (calculated with 50 mg of solids in 100 mL of 0.1 M NaCl solution). Reprinted with permission from Liu et al. 2019.[12] Copyright 2023 American Chemical Society.

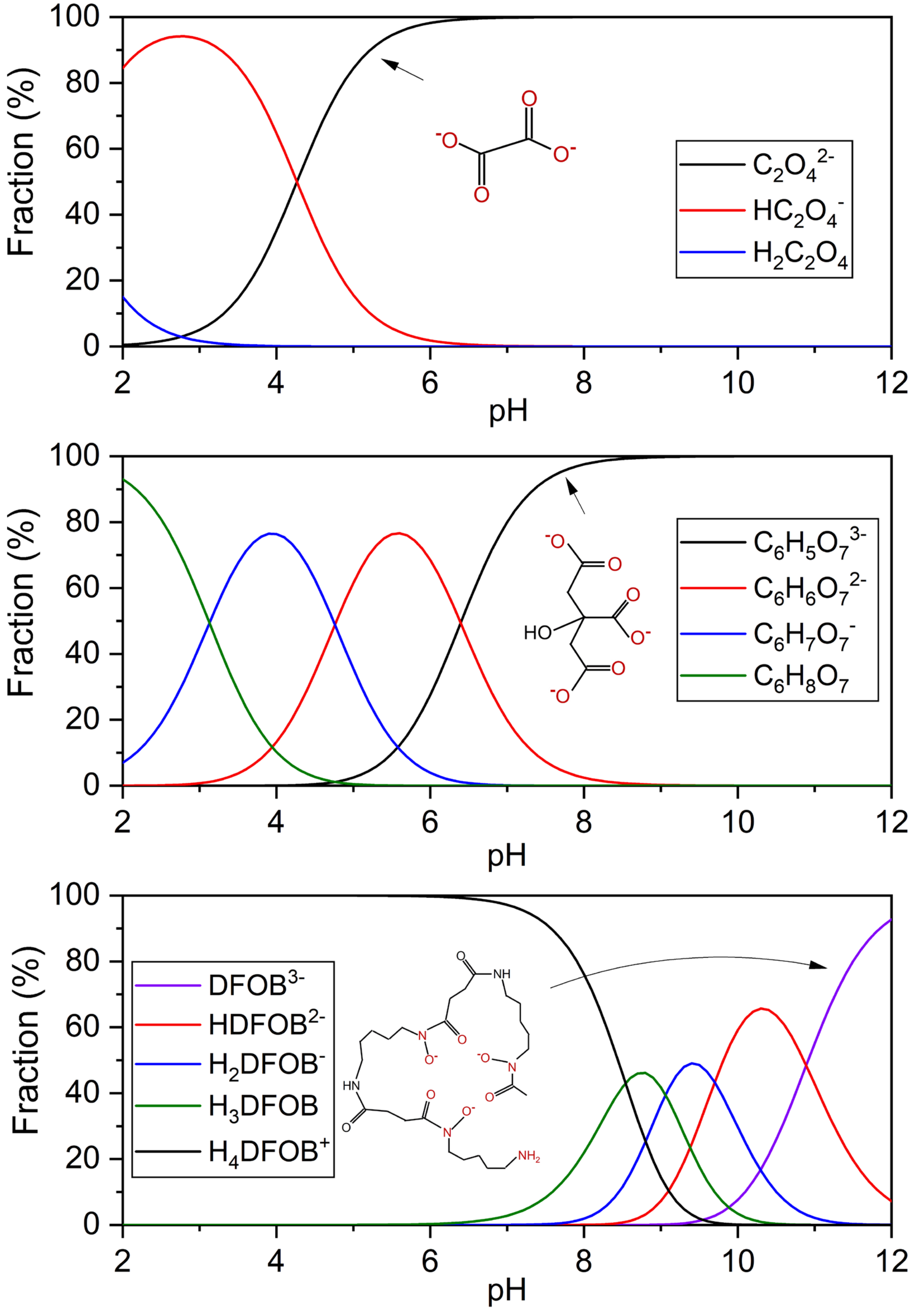

Figure S2. Speciation of oxalate, citrate, and DFOB as a function of pH. Oxalate: $pK_{a1} = 1.25$, $pK_{a2} = 4.27$;[6] Citrate: $pK_{a1} = 3.13$, $pK_{a2} = 4.76$, $pK_{a3} = 6.40$;[13] DFOB: $pK_{a1} = 8.54$, $pK_{a2} = 9.06$, $pK_{a3} = 9.70$, $pK_{a4} = 10.89$.[14] Insert structures are in the deprotonated forms.

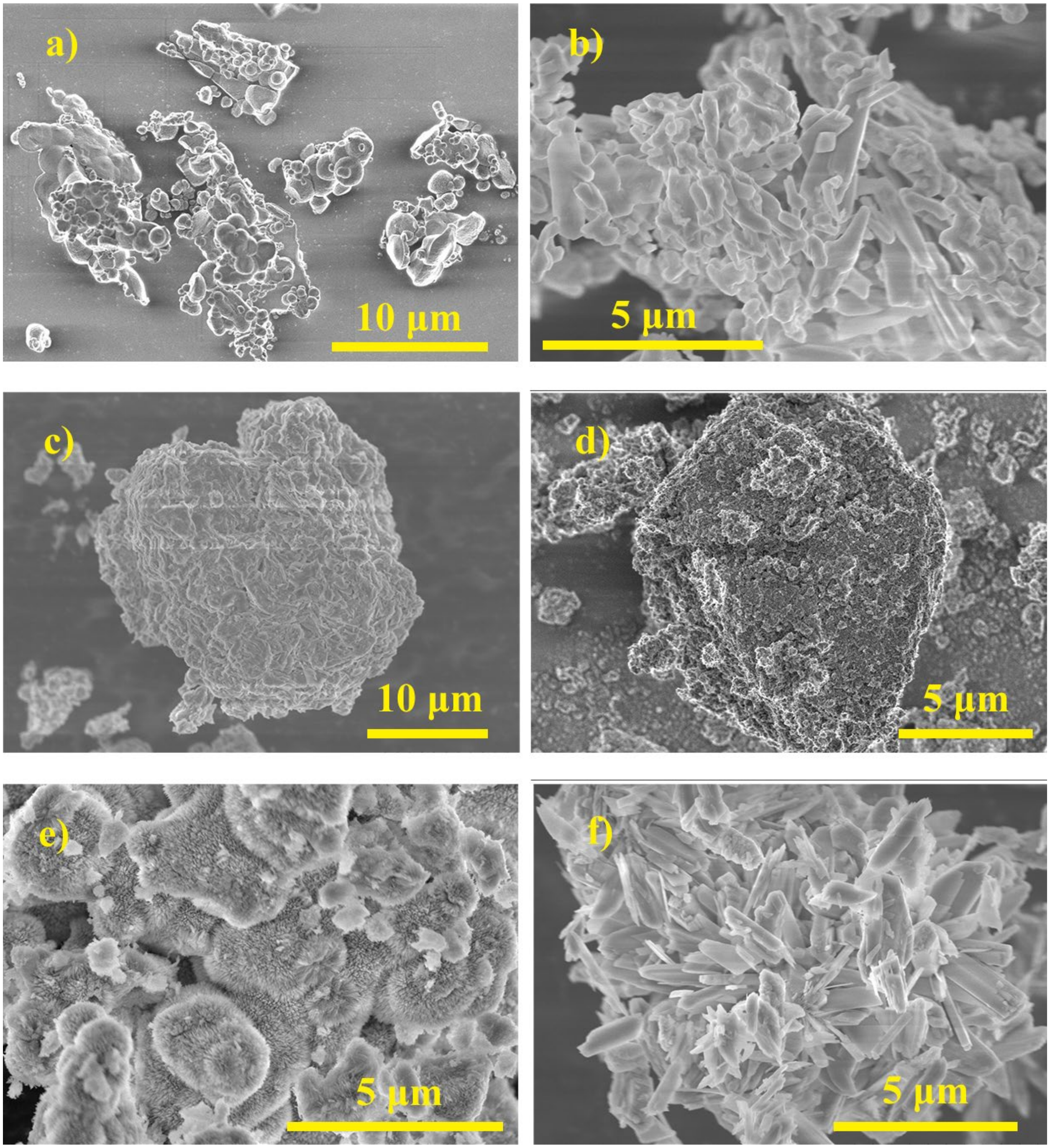

Figure S3. SEM images of REE minerals: **(a)** $La_2(CO_3)_3$, **(b)** $Nd_2(CO_3)_3$, **(c)** $Y_2(CO_3)_3$, **(d)** $LaPO_4$, **(e)** $NdPO_4$, and **(f)** YPO_4. More SEM images of $Y_2(CO_3)_3$ and YPO_4 can be found in our previous publication.[15]

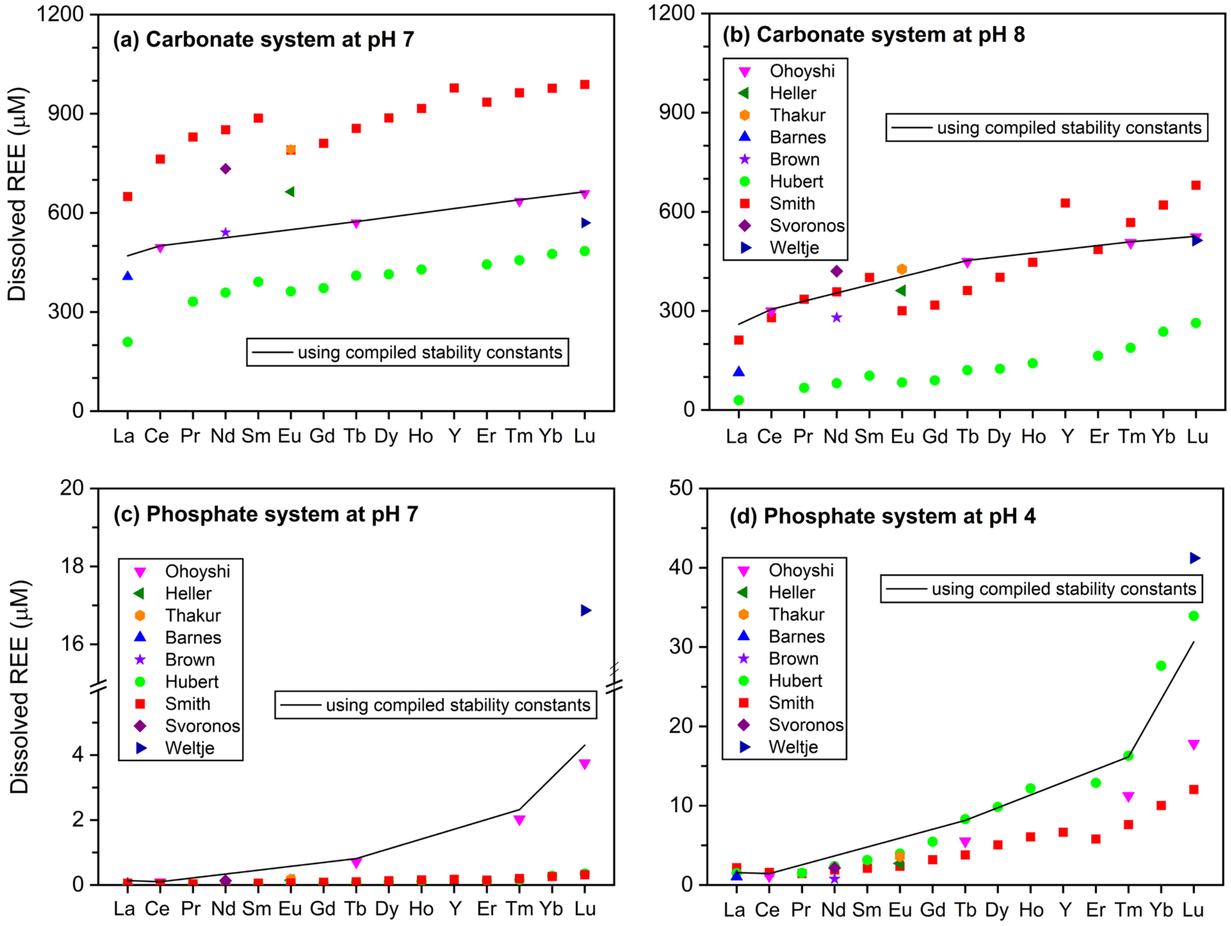

Figure S4. Comparison of PHREEQC modeling results for REE minerals in the presence of 1 mM citrate based on reported REE-citrate species and stability constants from literatures (colored symbols) and their compiled values used in this study (black line). Ohyoshi: Ref.[3]; Heller: Ref.[10]; Thakur: Ref.[9]; Barnes: Ref.[2]; Brown: Ref.[11]; Hubert: Ref.[4]; Smith: Ref.[6]; Svoronos: Ref.[5]; and Weltje: Ref.[7]

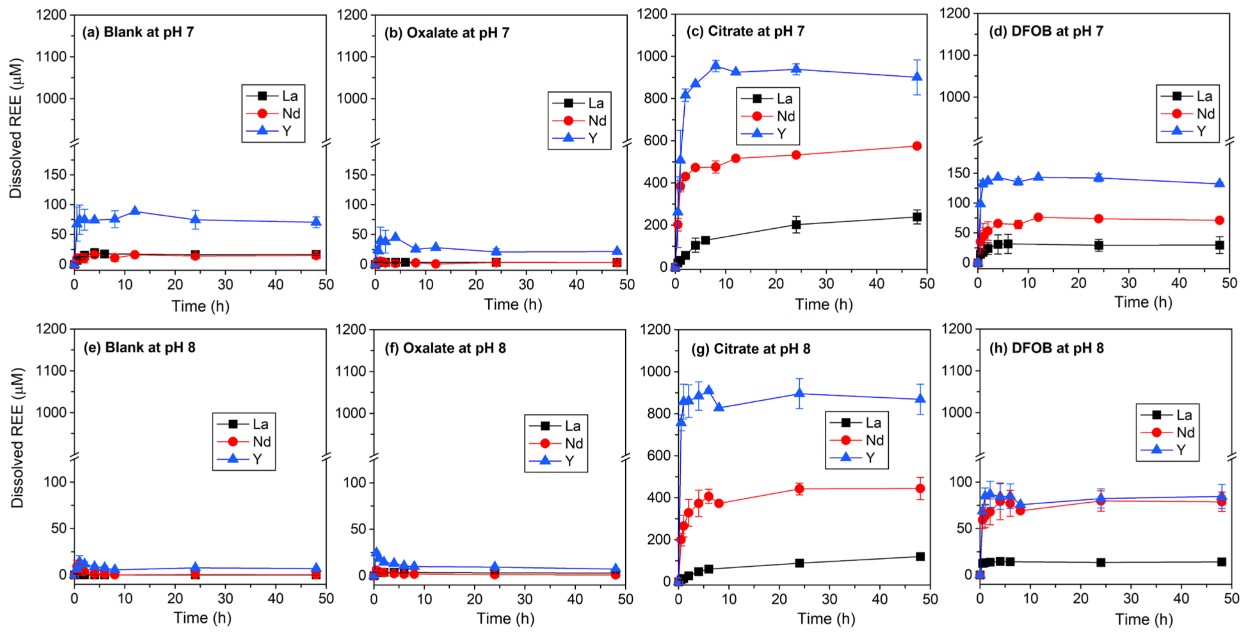

Figure S5. Time profiles of dissolved REE from $La_2(CO_3)_3$, $Nd_2(CO_3)_3$, and $Y_2(CO_3)_3$ in blank control, with 1 mM oxalate, 1 mM citrate, and 0.1 mM DFOB at **(a-d)** pH 7 and **(e-h)** pH 8. Note the breaks on y-axis.

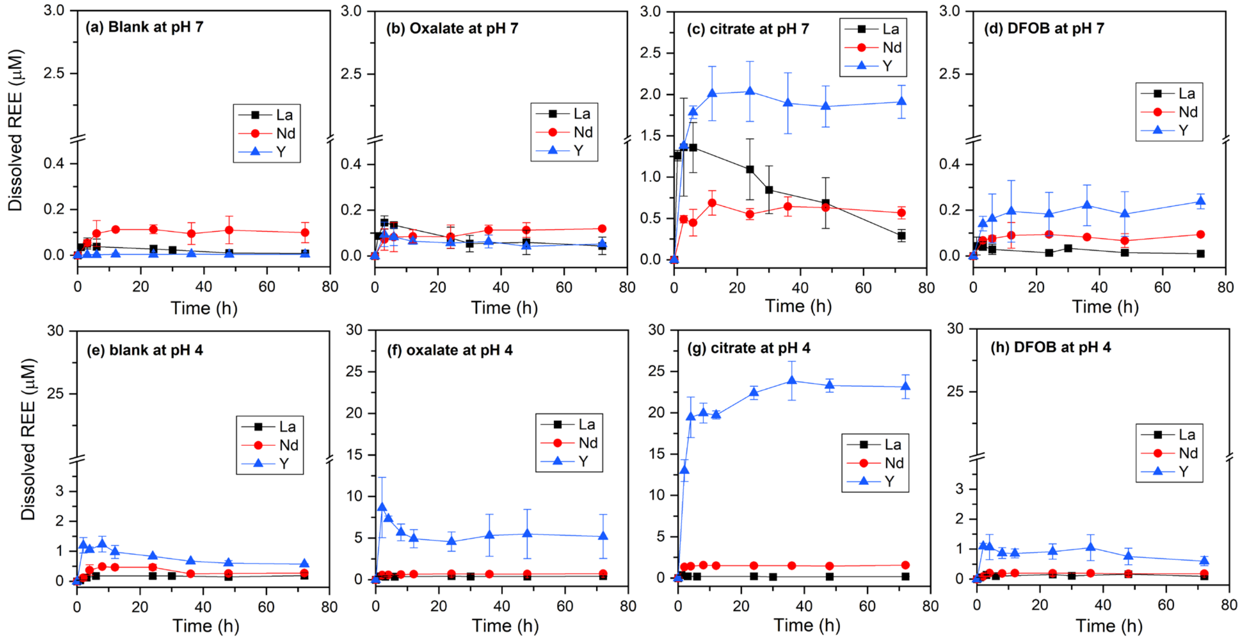

Figure S6. Time profiles of dissolved REE from $LaPO_4$, $NdPO_4$, and YPO_4 in blank control, with 1 mM oxalate, 1 mM citrate, and 0.1 mM DFOB at **(a-d)** pH 7 and **(e-h)** pH 4. Note the breaks on y-axis.

References

(1) Lucas, S.; Champion, E.; Bregiroux, D.; Bernache-Assollant, D.; Audubert, F. Rare earth phosphate powders $REPO_4 \cdot nH_2O$ (Re= La, Ce or Y)—Part i. Synthesis and characterization. *Journal of Solid State Chemistry* **2004**, *177* (4-5), 1302-1311.
(2) Barnes, J.; Bristow, P. Lanthanum citrate complexes in acid solutions. *Journal of the Less Common Metals* **1970**, *22* (4), 463-465.
(3) Ohyoshi, A.; Ohyoshi, E.; Ono, H.; Yamakawa, S. A study of citrate complexes of several lanthanides. *Journal of Inorganic and Nuclear Chemistry* **1972**, *34* (6), 1955-1960.
(4) Hubert, S.; Hussonnois, M.; Guillaumont, R. Mise en evidence de l'effet nephelauxetique dans un complexe citrique des elements de la serie 4f. *Journal of Inorganic and Nuclear Chemistry* **1973**, *35* (8), 2923-2944.
(5) Svoronos, D.-R.; Boulhassa, S.; Guillaumont, R.; Quarton, M. Citric complexes and neodymium citrate: NdCit, $3H_2O$. *Journal of Inorganic and Nuclear Chemistry* **1981**, *43* (7), 1541-1545.
(6) Smith, R.; Martell, A.; Motekaitis, R. NIST standard reference database 46. *NIST critically selected stability constants of metal complexes database Ver* **2004**, *2*.
(7) Weltje, L.; Verhoof, L. R.; Verweij, W.; Hamers, T. Lutetium speciation and toxicity in a microbial bioassay: Testing the free-ion model for lanthanides. *Environmental science & technology* **2004**, *38* (24), 6597-6604.
(8) Mathur, J.; Cernochova, K.; Choppin, G. Thermodynamics and laser luminescence spectroscopy of binary and ternary complexation of Am^{3+}, Cm^{3+} and Eu^{3+} with citric acid, and citric acid+ EDTA at high ionic strength. *Inorganica chimica acta* **2007**, *360* (6), 1785-1791.
(9) Thakur, P.; Xiong, Y.; Borkowski, M.; Choppin, G. R. Thermodynamic modeling of trivalent Am, Cm and Eu-citrate complexation in concentrated NaClO4 media. *Radiochimica Acta* **2012**, *100* (3), 165-172.
(10) Heller, A.; Barkleit, A.; Foerstendorf, H.; Tsushima, S.; Heim, K.; Bernhard, G. Curium (III) citrate speciation in biological systems: a europium (III) assisted spectroscopic and quantum chemical study. *Dalton Transactions* **2012**, *41* (45), 13969-13983.
(11) Brown, M. A.; Kropf, A. J.; Paulenova, A.; Gelis, A. V. Aqueous complexation of citrate with neodymium (III) and americium (III): a study by potentiometry, absorption spectrophotometry, microcalorimetry, and XAFS. *Dalton Transactions* **2014**, *43* (17), 6446-6454.
(12) Liu, P.; Huang, R.; Tang, Y. Comprehensive understandings of rare earth element (REE) speciation in coal fly ashes and implication for REE extractability. *Environmental science & technology* **2019**, *53* (9), 5369-5377.
(13) Parkhurst, D. L.; Appelo, C. Description of input and examples for PHREEQC version 3—a computer program for speciation, batch-reaction, one-dimensional transport, and inverse geochemical calculations. *US geological survey techniques and methods* **2013**, *6* (A43), 497.
(14) Christenson, E. A.; Schijf, J. Stability of YREE complexes with the trihydroxamate siderophore desferrioxamine B at seawater ionic strength. *Geochimica et Cosmochimica Acta* **2011**, *75* (22), 7047-7062.
(15) Liu, P.; Yang, L.; Wang, Q.; Wan, B.; Ma, Q.; Chen, H.; Tang, Y. Speciation transformation of rare earth elements (REEs) during heating and implications for REE behaviors during coal combustion. *International Journal of Coal Geology* **2020**, *219*, 103371.
